# Multitarget Hybrid Fasudil Derivatives as a New Approach to the Potential Treatment of Amyotrophic Lateral Sclerosis

**DOI:** 10.1021/acs.jmedchem.1c01255

**Published:** 2022-01-05

**Authors:** Olmo Martín-Cámara, Marina Arribas, Geoffrey Wells, Marcos Morales-Tenorio, Ángeles Martín-Requero, Gracia Porras, Ana Martínez, Giorgio Giorgi, Pilar López-Alvarado, Isabel Lastres-Becker, J. Carlos Menéndez

**Affiliations:** † Unidad de Química Orgánica y Farmacéutica, Departamento de Química en Ciencias Farmacéuticas, Facultad de Farmacia, Universidad Complutense, Plaza de Ramón y Cajal sn, 28040 Madrid, Spain; ‡ Instituto de Investigaciones Biomédicas “Alberto Sols” UAM-CSIC, Department of Biochemistry, School of Medicine, and Institute Teófilo Hernando for Drug Discovery, Universidad Autónoma de Madrid, 28029 Madrid, Spain; § UCL School of Pharmacy, University College London, 29/39 Brunswick Square, London WC1N 1AX, United Kingdom; ∥ Centro de Investigaciones Biológicas Margarita Salas, CSIC, Ramiro de Maeztu 9, 28040 Madrid, Spain; ⊥ Centro de Investigación Biomédica en Red de Enfermedades Neurodegenerativas (CIBERNED), Instituto de Salud Carlos III, 28031 Madrid, Spain

## Abstract

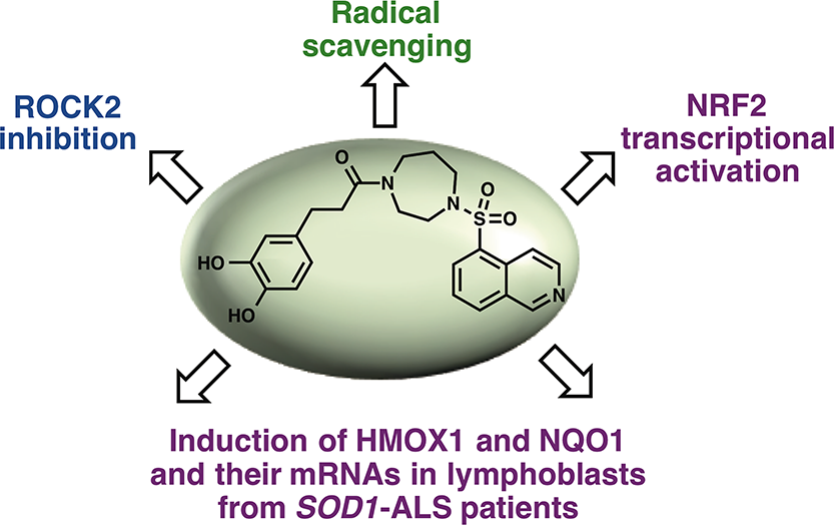

Hybrid compounds containing structural fragments of the Rho kinase inhibitor fasudil and the NRF2 inducers caffeic and ferulic acids were designed with the aid of docking and molecular mechanics studies. Following the synthesis of the compounds using a peptide-coupling methodology, they were characterized for their ROCK2 inhibition, radical scavenging, effects on cell viability (MTT assay), and NRF2 induction (luciferase assay). One of the compounds (**1d**) was selected in view of its good multitarget profile and good tolerability. It was able to induce the NRF2 signature, promoting the expression of the antioxidant response enzymes HO-1 and NQO1, via a KEAP1-dependent mechanism. Analysis of mRNA and protein levels of the NRF2 pathway showed that **1d** induced the NRF2 signature in control and *SOD1*-ALS lymphoblasts but not in sALS, where it was already increased in the basal state. These results show the therapeutic potential of this compound, especially for ALS patients with a *SOD1* mutation.

## 1 Introduction

Amyotrophic lateral sclerosis (ALS) is a degenerative disease that leads to the destruction of neuromuscular junctions of the first and second motoneurons (MNs), thereby causing progressive muscle weakness and atrophy accompanied by exaggerated tendon reflexes.^1,2^ About 35% of patients with ALS suffer behavioral or cognitive impairment, with an additional 15% having frontotemporal dementia.^3,4^ After a few years, this paralysis generally becomes lethal due to overall respiratory failure. The prevalence of ALS ranges from 2 to 5 cases per 100,000 and occurs sporadically (sALS) or in a familial form (fALS). The best-known cause of ALS, responsible for one-fifth of fALS cases, is a mutation in the gene encoding superoxide dismutase 1 (*SOD1*), an enzyme abundant in the cytoplasm and mitochondria of virtually all cell types. Although this mutation was the first to be identified,^5^ more recently, several pathogenic mutations related to ALS have been found, such as those in the TDP-43, FUS, and C9ORF72 proteins.^6^ On the other hand, it has been described that single nucleotide polymorphisms (SNPs) in the promoter region of NEF2L2, which increased NRF2 protein expression, were associated with a delayed disease onset of ALS. These results suggested that variations in *NEF2L2*, which encodes the master regulator of oxidative stress defense NRF2, may affect sALS progression.

Despite multiple clinical trials,^7,8^ to date, only the glutamate release inhibitor riluzole, the free-radical scavenger edaravone, and the tyrosine kinase inhibitor masitinib have been approved for the treatment of ALS, in the latter case for compassionate use.^9^ The effect of these drugs is very modest and prolongs the survival of patients by only a few months.^10^ The mechanisms of ALS pathogenesis^11^ involve multiple factors that include protein aggregation, oxidative stress, mitochondrial dysfunction, excitotoxicity, disturbance of selective autophagy pathways, degenerative processes related to neuron–glia interactions, alterations in RNA metabolism, cytoskeletal defects, and apoptosis. This suggests that, rather than addressing a single target, treatments of this disorder should be directed to different molecular pathways through a multidrug combination therapy.^12^

A common feature shared by ALS and several additional neurodegenerative disorders that affect voluntary muscle movement is the alteration of the activity of Rho GTPase,^13^ a protein that forms part of the Rho-ROCK signaling pathway and regulates the formation of the actin cytoskeleton in nerve cells. The inhibition of Rho kinases (ROCK), a family of serine/threonine kinases, using small molecule inhibitors, such as fasudil^14^ or Y-27632, may not only improve the regenerative response in the injured central nervous system (CNS) but also improve neuronal survival,^15−17^ including the promotion of neuromuscular junction maturation.^18^ For this reason, the U.S. Food and Drug Administration (FDA) has allowed fasudil to be tested in clinical trials for ALS^19^ and accepted its compassionate use in ALS patients.^20^

On the other hand, the role of oxidative stress, inflammation, and mitochondrial dysfunctions as important pathogenic mechanisms in ALS is well-established. Several antioxidant molecules and detoxifying enzymes are implicated in the defense against oxidative stress. The most important of these mechanisms is orchestrated by NRF2 (nuclear factor erythroid 2-related factor 2), the master regulator of cellular redox homeostasis.^21^ In nonstressed conditions, the N-terminal domain of the cap’n’collar homology (ECH)-associated protein 1 (KEAP1) presents NRF2 for ubiquitination by cullin 3 and RING-box protein1 (CUL3/RBX1)^22^ and subsequent degradation by the proteasome. In response to oxidative or electrophilic stressors, KEAP1 loses its ability to repress NRF2 due to modification of critical cysteines, leading to NRF2 stabilization and activation of its transcriptional activity.^23,24^ An alternative mechanism of regulation of NRF2 stability involves the phosphorylation of its Neh6 domain mediated by glycogen synthase kinase 3 (GSK-3), which initiates the recruitment of the β-transducin repeat-containing protein (β-TrCP) and facilitates the interaction between NRF2 and the CUL1/RBX1 complex for ubiquitin-proteasome degradation of NRF2.^25^ In the nucleus, NRF2 dimerizes mainly with the cognate bZip partners MAF G, K, and F and then binds to the antioxidant response element (ARE) activating the transcription of cytoprotective genes including several antioxidant and anti-inflammatory enzymes, which makes this pathway an increasingly important target in neurodegenerative diseases.^26^ In relation to ALS, it has been shown that NRF2 mRNA and protein levels were reduced in ALS patients relative to control tissues,^27,28^ although NRF2 target genes were not analyzed. Recent studies performed in our laboratory in ALS patient-derived lymphoblasts, which recapitulate features of affected MNs,^29^ clearly demonstrated that NRF2 activity appears to be differentially regulated in sALS or *SOD1*-ALS.^30^ These data indicate that pharmacological modulation of NRF2 as a therapeutic strategy for ALS should be personalized according to the molecular differences displayed by the patient. Indeed, NRF2-activating compounds have demonstrated therapeutic efficacy in *SOD1* mouse models of ALS.^31^

**Figure 1:**
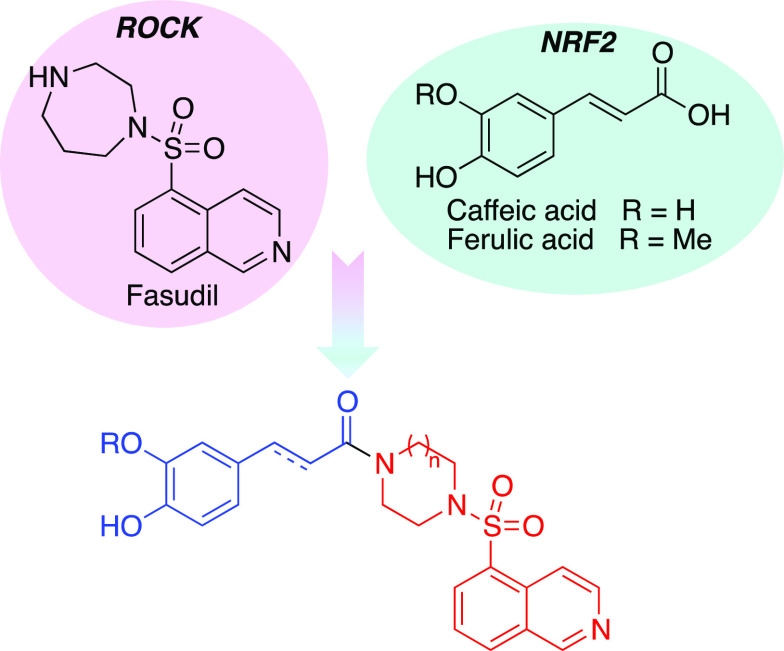
Our molecular hybridization strategy.

In the context of the multitarget-directed ligand (MTDL) approach, i.e., the purposeful design of small molecules able to inhibit several pathological mechanisms, we have focused on preventing damage triggered by oxidative stress through NRF2 activation while simultaneously avoiding the pathological consequences of ROCK overphosphorylation using a polypharmacology approach. To this end, we have hybridized the ROCK inhibitor fasudil with two natural products (caffeic and ferulic acids) capable of inducing the activation of NRF2 that also have radical scavenging properties due to their phenolic nature (Figure 1).

While multitarget approaches are becoming increasingly popular in drug discovery against multifactorial diseases,^32^ their application to ALS therapy has received little attention. The peptidic drug alirinetide (GM604, GM6, l-phenylalanyl-l-seryl-*N*^5^-(diaminomethylene)-l-ornithyl-l-tyrosyl-l-alanyl-*N*^5^-(diaminomethylene)-l-ornithine), which was granted fast track status by the FDA and orphan drug designation by the EMA and has undergone phase II clinical trials for the treatment of ALS,^33^ is believed to promote neuron survival via a multitargeted regulation of developmental pathways,^34^ although it was not designed using the MTDL paradigm. Regarding small molecules, the multitarget iron chelator VAR10303 has shown beneficial effects on ALS mice.^35^ In this context, we describe here the design and synthesis of a small library of fasudil–ferulic/caffeic hybrid compounds and their characterization as an NRF2 signaling inducer and their therapeutic potential, especially for ALS patients with a *SOD1* mutation.

## 2 Results and Discussion

### 2.1 Compound Design

**Figure 2:**
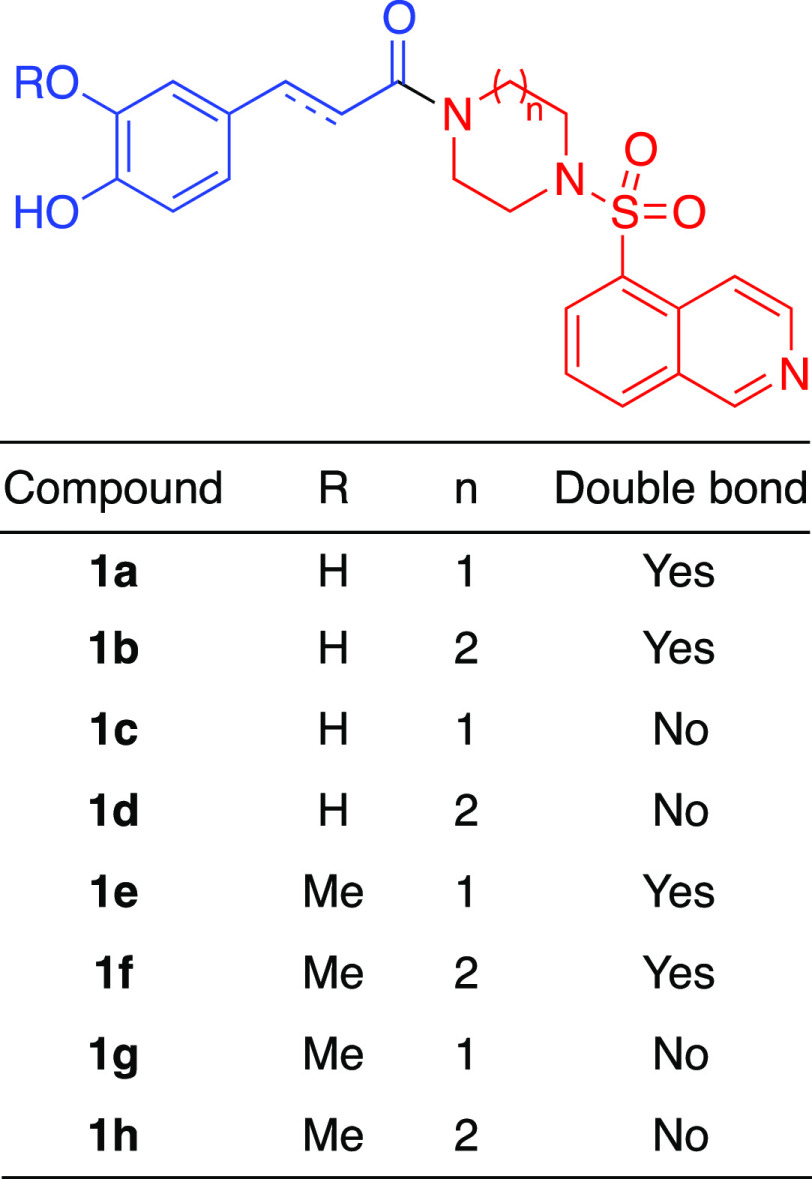
Structures of compounds **1** studied in this article.

The fasudil-based hybrid compounds studied here are shown in Figure 2. Together with the cinnamic acid derivatives, we also planned the preparation of the corresponding dihydro derivatives, to establish the relevance of the double bond on NRF2 induction. To our knowledge, there has been only one precedent of a multitarget drug designed from fasudil, in which it was coupled to an antioxidant (lipoic acid); this compound proved to be less cytotoxic than other fasudil derivatives due to the protective effect of the second structural fragment.^36^

First, computational ADME studies were carried out using SwissADME.^37^ For all compounds, a high gastrointestinal absorption, important for oral bioavailability, was predicted. Moreover, no compound showed violations of Lipinski’s rule of five. The details of this study are shown in Table S1 (see the Supporting Information). A single PAINS alert appeared in some of the compounds due to the presence of catechol moieties. In order to discard false positives by nonspecific target binding, we studied the activity of compound **1d** as an inhibitor of the kinase GSK-3β and also its ability to reduce aberrant TDP-43 phosphorylation or TDP-43 expression in lymphoblasts from ALS patients (see the details in the Supporting Information, Figure S1). The negative results of all these experiments allow us to discard indiscriminate binding of **1d** to biological targets. On the other hand, a complementary study performed with ADMETLab 2.0^38^ gave less favorable results in terms of oral absorption and raised some toxicity concerns that will need to be addressed in future optimization efforts (Table S2).

We also assessed computationally whether our planned structural manipulation of fasudil would maintain affinity for the ROCK enzyme. Two main isoforms of the enzyme (ROCK1 and ROCK2) are known, with ROCK2 being the predominant form expressed in smooth muscles and the brain. For this reason, a crystal of the ROCK2 isoform (PDB 4WOT)^39^ was selected to perform the docking studies of compounds **1**. In the case of fasudil, which was studied in the first place in order to validate the docking protocol, all the critical interactions previously established by X-ray crystallography,^40^ i.e., a hydrogen bond with Met172 of the hinge region, and nonpolar interactions with some other residues in the hydrophobic pocket (Leu221, Ala231, Val106, and Met169) were located (Figure 4A). In the case of compounds **1**, similar interactions were found, together with some distant interactions of the catechol moiety with the protein (Leu123 and Phe136 residues). The binding energies of the whole family of compounds **1** were studied with Autodock Vina. As shown in Table 1, most of the compounds show similar or higher binding energies than the reference compound fasudil. Moreover, a careful examination of the energies reveals that the dihydro ligands (**1c**, **1d**, **1g**, and **1h**) show in all cases lower energies than the corresponding unsaturated compounds (**1a**, **1b**, **1e**, and **1f**). This behavior can be explained due to an increase in the degrees of freedom in the side chain of the dihydro ligands, which allows the catechol moiety to accommodate better to the distant region (Leu123 and Phe136).

**Table 1:** Free-Energy Estimation (kcal/mol) for the Complexes Formed by Compounds **1** and ROCK2

compound	energy (kcal/mol)
fasudil	–8.00
**1a**	–7.70
**1b**	–8.80
**1c**	–8.20
**1d**	–10.30
**1e**	–7.80
**1f**	–8.10
**1g**	–8.50
**1h**	–9.37

**Figure 3:**
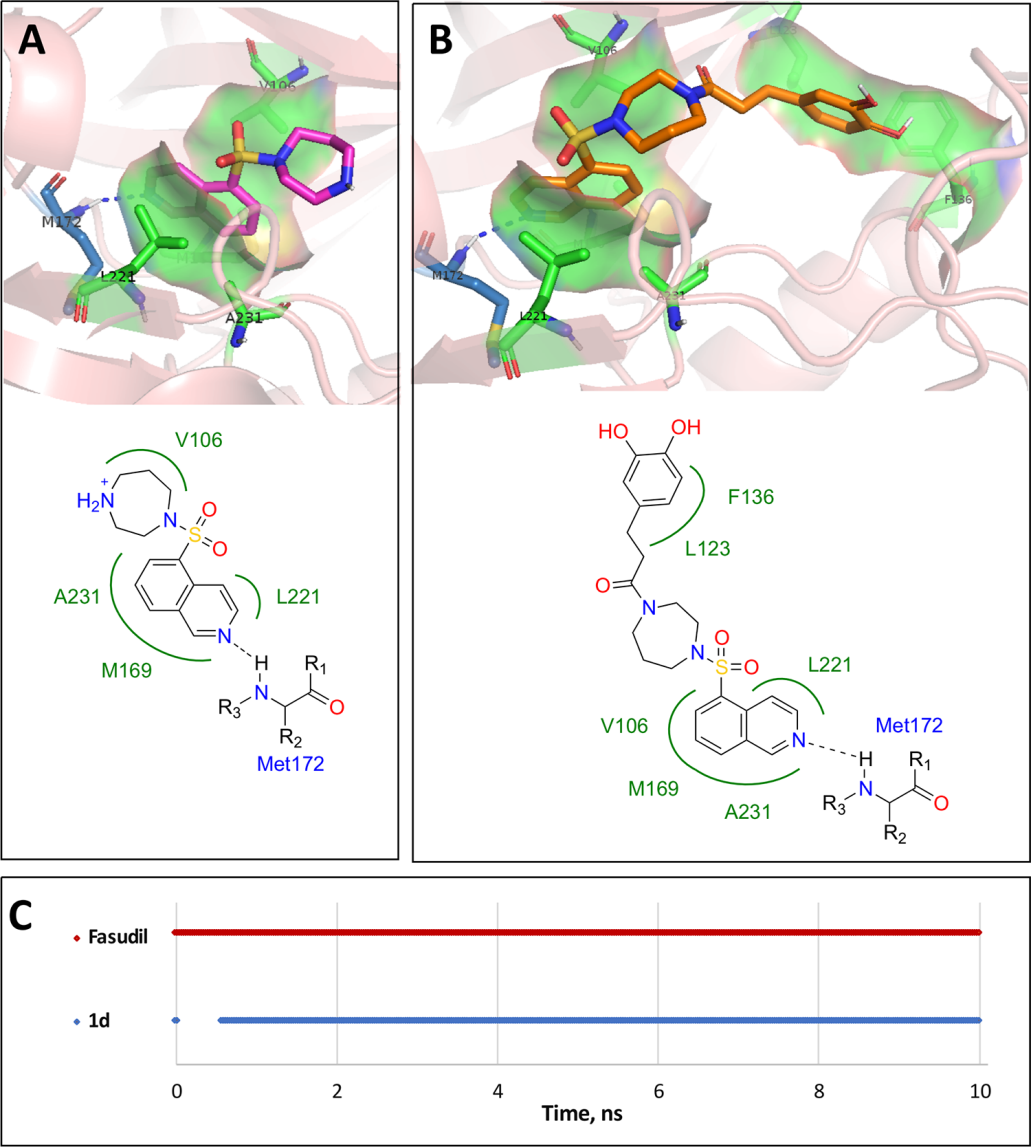
Docking and molecular dynamics studies on ROCK2. (A) Fasudil establishes a hydrogen bond with Met172, which can be seen as a dashed blue line. Also, there are some hydrophobic interactions with other close residues (Val106, Met169, Leu221, or Ala231), which can be seen as a green surface around the ligand. (B) Compound **1d** interacts with Met172 via a hydrogen bond, which is represented by a dashed blue line. There are also some hydrophobic interactions with other residues around the isoquinoline moiety (Val106, Met169, Leu221, or Ala231), and some distant residues interact with the catechol moiety (Leu123 and Phe136), which can be seen as a green surface around the ligand. (C) Timeline of the formation of the hydrogen bond (N(ligand)-H-Met172) for the interactions of the protein with fasudil or compound **1d**. Each point represents a snapshot taken when *d*[N(**1d**)–H(Met172)] is below 2.5 Å.

To further analyze the stability of the system and the different binding energies, molecular dynamics simulations (10 ns) and subsequent metadynamics were performed on fasudil and compound **1d** (Figure 3). Both fasudil and **1d** gave stable complexes with the enzyme along the simulation trajectory. To determine the stability of the complexes of fasudil or **1d** with the enzyme along the simulation trajectory, we calculated the root-mean-square deviation (RMSD) of the ligand structure compared with the values obtained after energy minimization for each snapshot of the simulation. In the case of fasudil, the initial conformation remained very stable, with an RMSD value of 1.77 ± 0.35 Å. In the case of **1d**, the compound showed an RMSD value of 2.18 ± 0.24 Å, which is less stable than fasudil but still considered stable. We also determined the stability of the hydrogen bond of the ligand with the hinge region by measuring the evolution of the distance between the isoquinoline nitrogen and the backbone nitrogen-bonded hydrogen of Met172 (*d*[N(ligand)–H(Met172)]) for each snapshot of the simulation. The average hydrogen bonding distance for fasudil was 2.02 ± 0.17 Å, and it remained below 2.5 Å nearly all the time (98.8%). On the other hand, *d*[N(**1d**)–H(Met172)] was 2.19 ± 0.56 Å on the average, and it remained below 2.5 Å for 90.8% of the time.

The binding energy for each pose was calculated over 200 snapshots of the complex from the final 2 ns of simulation. The free energy of binding was obtained by using the molecular mechanics Poisson–Boltzmann surface area (MM-PBSA) approach, which calculates the final energy from terms corresponding to potential energy in vacuum (van der Waals energy and electrostatic energy) and solvation energies (including polar and nonpolar terms).^41^ The results of these calculations are given in Table 2.

**Table 2:** Comparison of the Binding of Fasudil and Compound **1d** to ROCK2^a^

energy	fasudil–ROCK2	**1d**–ROCK2
Δ*E*_VdW_	–144.9 ± 8.6	–140.5 ± 10.3
Δ*E*_Elec_	–17.1 ± 6.9	–60.3 ± 22.0
Δ*E*_Polar_	86.4 ± 12.2	108.3 ± 30.7
Δ*E*_Nonpolar (SASA)_	–15.9 ± 0.6	–15.6 ± 1.1
Δ*G*_Binding_	–91.5 ± 11.2	–108.2 ± 16.8

a Energies are given in kJ/mol.

After the assessment of the computational stability of the complex of compound **1d** and ROCK2, we concluded that our planned introduction of the cinnamic/dihydrocinnamic side chains is in principle compatible with ROCK2 inhibition.

### 2.2 Synthesis

**Scheme 1:**
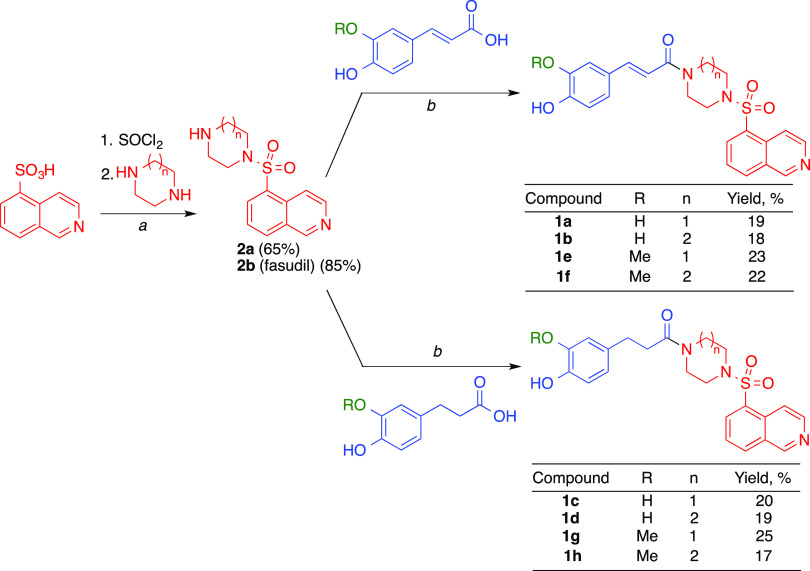
Synthesis of Compounds **1** with Reagents and Conditions: (*a*) Cl_2_SO, DMF, 70 °C, and 2 h; (*b*) EDCI, HOBt·H_2_O, Et_3_N, THF, 4 °C, and 72 h

The starting materials **2** were prepared from isoquinoline-5-sulfonic acid and piperazine or homopiperazine using a literature method.^42^ Their treatment with caffeic (R = H) acid or ferulic (R = Me) acid using a combination of 1-ethyl-3-(3-dimethylaminopropyl)carbodiimide (EDCI) and 1-hydroxybenzotriazole (HOBt) as coupling reagents afforded compounds **1a**,**b**,**e**,**f** in moderate yields. A similar treatment with dihydrocaffeic and dihydroferulic acid furnished phenylpropionamide derivatives **1c**,**d**,**g**,**h** (Scheme 1).

### 2.3 Rho Kinase Inhibition Studies

Because ROCK2 predominates in the human brain,^43^ we focused on the ability of compounds **1** to inhibit this isoform of the enzyme. As summarized in Table 3, we measured IC_50_ values for all compounds and also for fasudil (**2b**), and these data led to the conclusion that piperazine derivatives are generally less active as ROCK2 inhibitors than their homopiperazine counterparts (**1a** > **1b**, **1c** > **1d**, and **1e** > **1f**), catechols are more active than *O*-methylcatechols (**1a** > **1e**, **1b** > **1f**, **1c** > **1g**, and **1d** > **1h**), and the double bond generally favors ROCK2 inhibition (**1a** < **1c**, **1b** < **1d**, and **1e** > **1f**). From these studies, compounds **1a**, **1c**, and **1d** were shown to inhibit ROCK2 with a potency similar to the reference compound. These experimental data are in good agreement with the computational studies, which predict a similar or higher free energy for the binding of catechol derivatives in comparison to their *O*-methyl derivatives and also a better binding of the dihydro derivatives.

**Table 3:** ROCK2 Inhibition by Compounds **1** and the Reference Compound Fasudil, Expressed as IC_50_ Values (μM)

compound	IC_50_ (μM)	SD
fasudil	0.37	0.08
**1a**	0.79	0.25
**1b**	2.13	0.4
**1c**	0.32	0.09
**1d**	0.73	0.13
**1e**	2.33	0.78
**1f**	2.61	0.28
**1g**	>10	n/a
**1h**	0.90	0.11

### 2.4 Radical Scavenging Capacity

As mentioned in the Introduction, oxidative stress is an important factor in the progression of ALS, and thus, we tested the potential antioxidant effect of our compounds **1** as direct ROS scavengers. DPPH (2,2-diphenyl-1-picrylhydrazyl hydrate) is a stable free radical that is reduced in the presence of antioxidant molecules, giving a colorless solution. The DPPH reduction antioxidant assay was employed to study compounds **1**, finding that, as expected in view of their phenol functional groups, they have a good radical scavenging capacity. The DPPH reducing activity of our compounds was similar to those of caffeic and ferulic acids, which were used as references for the assay. The catechol derivatives **1a**–**d** were more effective as free-radical scavengers than their *O*-methyl counterparts **1e**–**h** (Table 4).

**Table 4:** Radical Scavenging Activity of Compounds **1**, Expressed as EC_50_ Values (DPPH Method)^a^

compound	EC_50_ (μM)	SD
caffeic acid	12.13	1.03
**1a**	9.93	1.18
**1b**	10.02	0.96
**1c**	10.11	0.69
**1d**	9.68	0.66
ferulic acid	22.83	0.69
**1e**	6.42	0.47
**1f**	9.61	0.63
**1g**	10.30	0.63
**1h**	12.00	0.99

a Caffeic and ferulic acids were employed as references.

### 2.5 Experimental ADME Studies

In order to extend the above-mentioned computational ADME profile, we have studied experimentally some *in vitro* experimental properties of compound **1d**, which was our best candidate for further optimization, as shown in subsequent sections. Most of the therapeutic compounds are biotransformed in the liver tissue, and therefore, liver microsomal fractions are widely used to study the *in vitro* metabolic stability in drug discovery phases. Compound **1d** was incubated with human microsomes, using as a control for the assay verapamil, a widely used drug with a well-known metabolism. The results obtained indicate that **1d** presents a better metabolic behavior than verapamil (Table 5), with a higher half-life (*t*_1/2_) and lower intrinsic clearance (CL_int_), which correspond to a longer time of compound exposure *in vivo*.

**Table 5:** Stability of Compound **1d** in Human Liver Microsomes

compound	*t*_1/2_ (min)	CL_int_^a^ (mL/min/mg protein)
**1d**	35.7	15.3
verapamil	20.2	26.9

a CL_int_, intrinsic clearance.^44^

**Figure 4:**
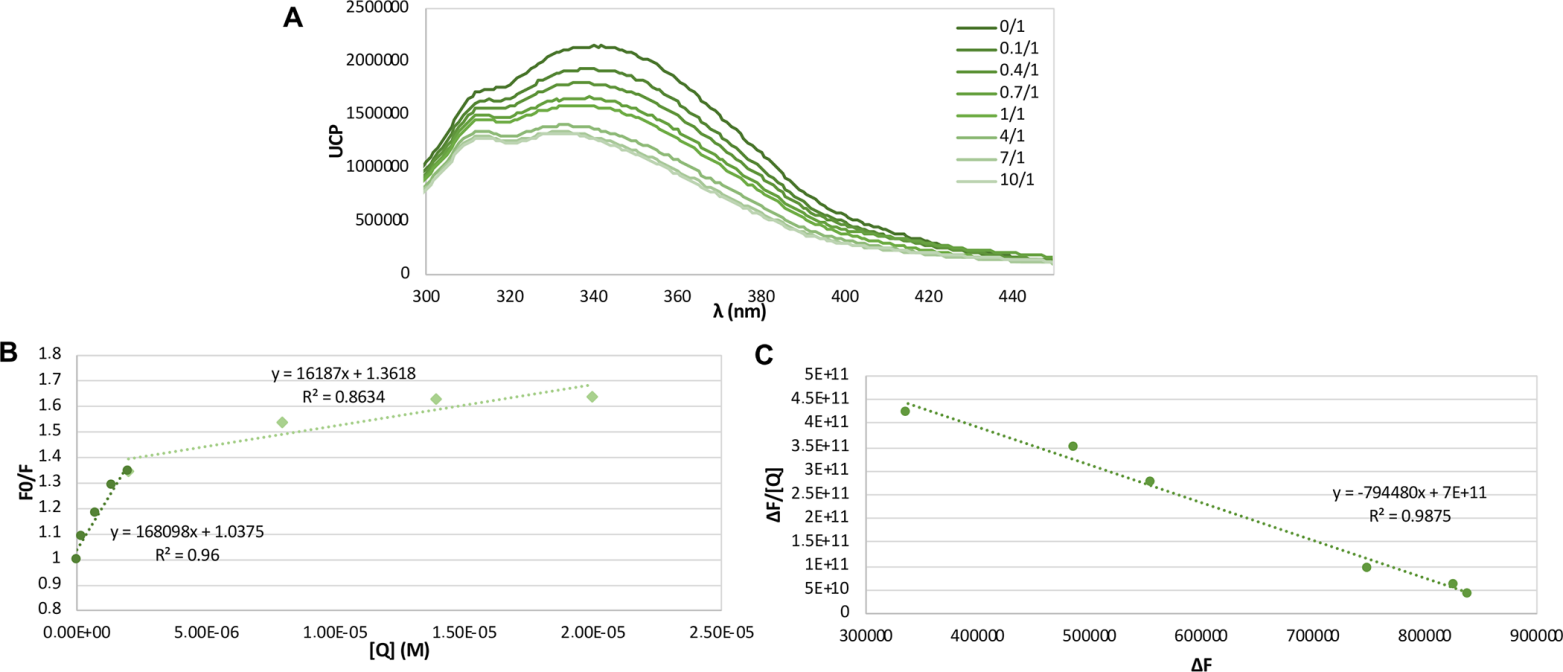
Interaction of compound **1d** with human serum albumin (HSA). (A) Emission spectra of HSA (2 μM) with increasing amounts of compound **1d** (λ_ex_ = 280 nm) in buffer of phosphate-buffered saline (PBS, 50 mM). The relation shown in the legend represents the ratio Q/HSA. (B) Stern–Volmer graphic representation of the fluorescence of HSA (2 μM) (λ_ex_ = 280 nm, λ_em_ = 337 nm) in the presence of increasing concentrations of compound **1d** (from 0 to 20 μM). In dark-green round markers are data from lower molar ratios, from 0/1 to 1/1 (Q/HSA), which fit with a linear distribution (*r*^2^ = 0.96). In light-green rhomboid markers are data from higher molar ratios, from 1/1 to 10/1 (Q/HSA), which do not fit with a linear distribution (*r*^2^ = 0.86). (C) Graphical representation of the Scatchard equation. The slope of the curve corresponds with −*K*_a_ (*K*_a_ = 7.94 × 10^5^ L mol^–1^).

Protein binding is another important parameter related to the ADME profile. In particular, the binding of drugs to serum albumin is the key to their distribution in the body, either by influencing the effective concentration of the drug at its site of action, since only an unbound drug is able to reach its target, or by changing the rate at which the drug is eliminated by interference with its glomerular filtration. For this reason, we studied the binding of compound **1d** to human serum albumin (HSA). It is well-known that the fluorescence quenching effect is an indirect method for studying the binding of small molecules to proteins such as HSA,^45,46^ and Figure 4 shows that the addition of increasing amounts of compound **1d** to a solution of human serum albumin (HSA) diminished the intensity of the fluorescence of the protein (λ_ex_ = 280 nm, λ_em_ = 337 nm). The quenching effect is evident at lower ratios, from 0/1 to 1/1 (**1d**/HSA), but at higher molar ratios, this effect diminished, suggesting that the system becomes saturated. In order to determine the nature of the quenching effect, the Stern–Volmer equation was applied, showing a linear behavior until the molar ratio reaches 1/1 (Q/HSA). From that point, the representation is not linear, which also suggests saturation effects. These data prove that compound **1d** binds to HSA generating a quenching effect defined by the constant *K*_SV_ = 1.68 × 10^5^ L mol^–1^. In order to determine the affinity of the binding, the Scatchard equation^45^ was employed, establishing that the **1d**·serum albumin complex has an association constant *K*_a_ = 7.94 × 10^5^ L mol^–1^. It can be concluded that **1d** binds to HSA with an affinity that is in the same order of magnitude as those of many well-established drugs.^47^

### 2.6 Effect of Compounds **1** on Cell Viability

*In vitro* cytotoxicity testing gives essential information for safety assessment and screening and for ranking compounds. The cytotoxic effect of these compounds was evaluated against HEK293T cells using the 3-(4,5-dimethylthiazol-2-yl)-2,5-diphenyltetrazolium bromide (MTT) assay. The concentrations used for each compound were 20 and 60 μM. In general, all compounds show toxicities below 20% (Table 6), except for compound **1e**, which shows significant cytotoxic effects (cell viability reduced to 61.85%). In the aggregate, these results indicate that, with the exception of **1e**, compounds **1** have a good tolerability.

**Table 6:** Analysis of Cell Viability of the Compounds **1**, Determined by MTT Assay^a^

compound	20 μM	60 μM
basal	99.987 ± 2.023	
DMF	90.359 ± 6.958	92.207 ± 0.462
**1a**	90.937 ± 1.634	100.680 ± 0.999
**1b**	96.873 ± 1.130	84.663 ± 1.827
**1c**	103.727 ± 1.204	104.793 ± 0.434
**1d**	100.427 ± 1.024	103.743 ± 0.837
**1e**	60.530 ± 2.735	55.927 ± 1.079
**1f**	93.913 ± 2.273	77.863 ± 3.899
**1g**	93.678 ± 2.202	87.540 ± 2.324
**1h**	99.383 ± 1.782	88.213 ± 2.434
fasudil	92.070 ± 3.714	83.434 ± 0.715

a HEK293T cells were treated with dimethyl fumarate (DMF) (20 or 60 μM) as a positive control of the NRF2 activator or two different concentrations of compounds **1** (20 and 60 μM) for 16 h. MTT experiments were performed in triplicates at least twice.

### 2.7 Compounds **1b**, **1c**, and **1d** Lead to NRF2 Transcriptional Activation

**Figure 5:**
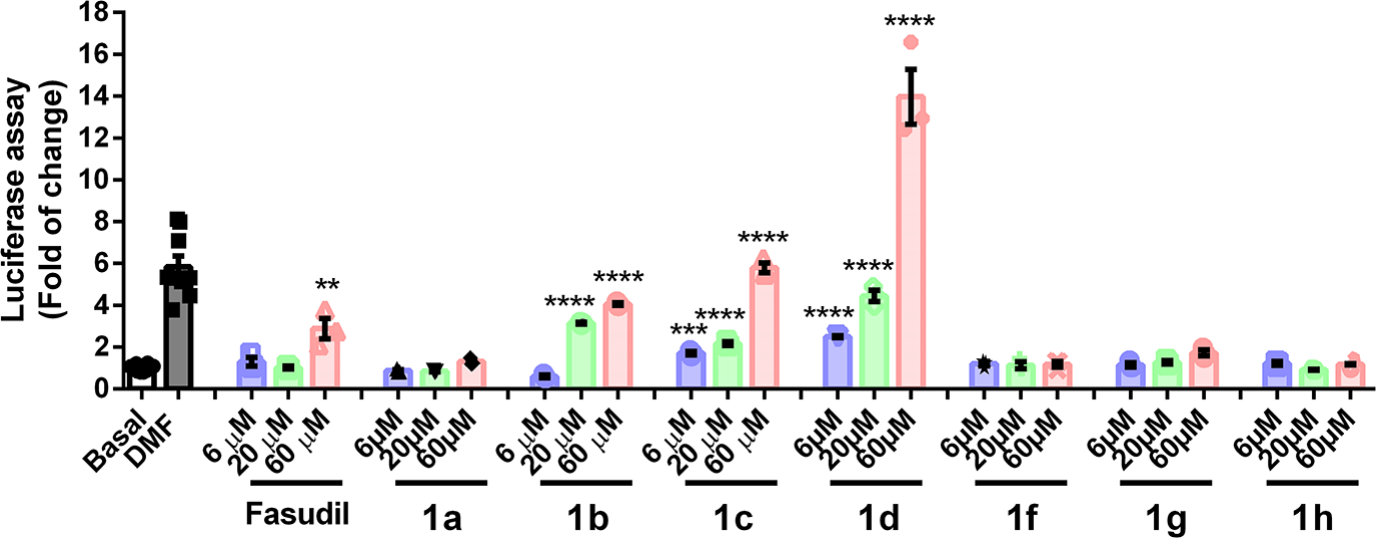
Compounds **1b**, **1c**, and **1d** showed a dose–response activation of the NRF2 reporter. HEK293T cells were cotransfected with the 3×ARE-LUC reporter and the Renilla control vector and treated with DMF (20 μM) as a positive control or three different concentrations of compounds **1** (6 μM in blue, 20 μM in green, and 60 μM in pink) for 16 h. Luciferase experiments were performed in triplicates at least twice. The values in graphs correspond to the mean ± S.E.M. To assess differences between groups, one-way ANOVA followed by a Tukey’s multiple comparison post-test was performed. Asterisks denote statistically significant differences with ***p < 0.001 and ****p < 0.0001. The numerical data corresponding to this figure can be found in Table S3.

To determine whether compounds **1** could activate NRF2 and induce its transcriptional activity, we used two different approaches. First, we performed a luciferase reporter assay using a promoter that contains three ARE sites in tandem (3×ARE-LUC).^48^ Dimethyl fumarate (DMF) (20 μM), a well-known inducer of NRF2, was used as a positive control. We found a dose-dependent activation of the NRF2 reporter by **1b**, **1c**, and **1d** (Figure 5). In the case of compound **1b**, the activation of NRF2 could be due to the cytotoxic effect that we have observed previously.^49^ Compound **1d**, at a dose of 20 μM, had a similar effect to the positive DMF control at the same dose.

The low activity of fasudil confirmed that NRF2 reporter induction was mainly due to the presence of the cinnamic or dihydrocinnamic side chain. It is relevant to note that the three active compounds were catechol derivatives and that the double bond in the side chain does not seem to have an important role in activity since the more potent compounds **1c** and **1d** lack this structural feature.

Multitarget-directed ligands are generally considered to have a balanced activity profile if their potency ratio between any two targets is not higher than 10.^50^ In order to study this aspect of our compounds, we have determined the values of CD (i.e., the concentration able to duplicate the response to the luciferase assay relative to basal) for those that showed activity in this regard, namely, **1b**–**1d**. The results are shown in Table 7, together with the IC_50_ values for ROCK2 inhibition and the corresponding ratios. While compound **1c** showed an unbalanced profile, both **1b** and **1d** were successful in this regard. In particular, our hit compound **1d** showed a ratio of 1.4 between NRF2 induction and ROCK2 inhibition, which can be regarded as a very well-balanced profile and a good starting point for future optimization efforts.

**Table 7:** Comparison of NRF2 Inducing Capacity of Compounds **1b**–**d**, Expressed as CD (Concentration Needed to Double Luciferase Expression), and Their ROCK2 Inhibition Activity, Measured by IC_50_ Values, Showing that Compounds **1b** and **1d** Have a Well-Balanced Multitarget Profile

compound	NRF2 induction, CD (μM)	ROCK2 inhibition, IC_50_ (μM)	NRF2/ROCK2 ratio
**1b**	12	2.13	5.2
**1c**	15	0.32	46.9
**1d**	1	0.73	1.4

**Figure 6:**
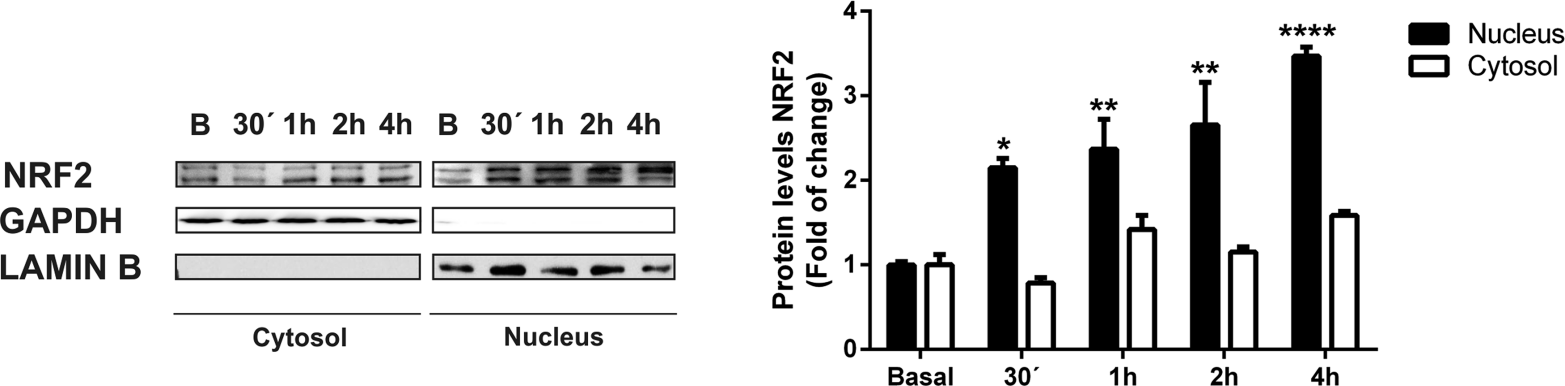
Compound **1d**-induced nuclear translocation of NRF2. SH-SY5Y cells were incubated in the presence of compound **1d** (20 μM) for 1, 2, and 4 h, and subcellular fractionations were analyzed by immunoblotting: upper panel, NRF2 levels; middle panel, GAPDH levels used as a cytosol protein loading control; lower panel, Lamin B level used as a nuclear protein loading control. Densitometric quantification of NRF2 protein levels of representative blots. Experiments were performed in duplicate at least twice. To assess differences between groups, one-way ANOVA followed by a Tukey’s multiple comparison post-test was performed. Asterisks denote statistically significant differences with *p < 0.05, **p < 0.01, and ****p < 0.0001.

To carry out the second approach, i.e., subcellular fractionation, we selected compound **1d**, which had no adverse effects on cell viability and also induced the expression of 3×ARE-LUC in a dose-dependent manner, in a similar concentration range to DMF. We analyzed the subcellular distribution of NRF2 in SH-SY5Y cells after treatment with compound **1d** at different time points. As shown in Figure 6, subcellular fractionation assays demonstrated that compound **1d** induced a significant accumulation of NRF2 in the nucleus and, to a lesser extent, in the cytosol. These results showed that compound **1d** could induce NRF2 transcriptional activation.

### 2.8 Compound **1d** Activates the NRF2 Signature

**Figure 7:**
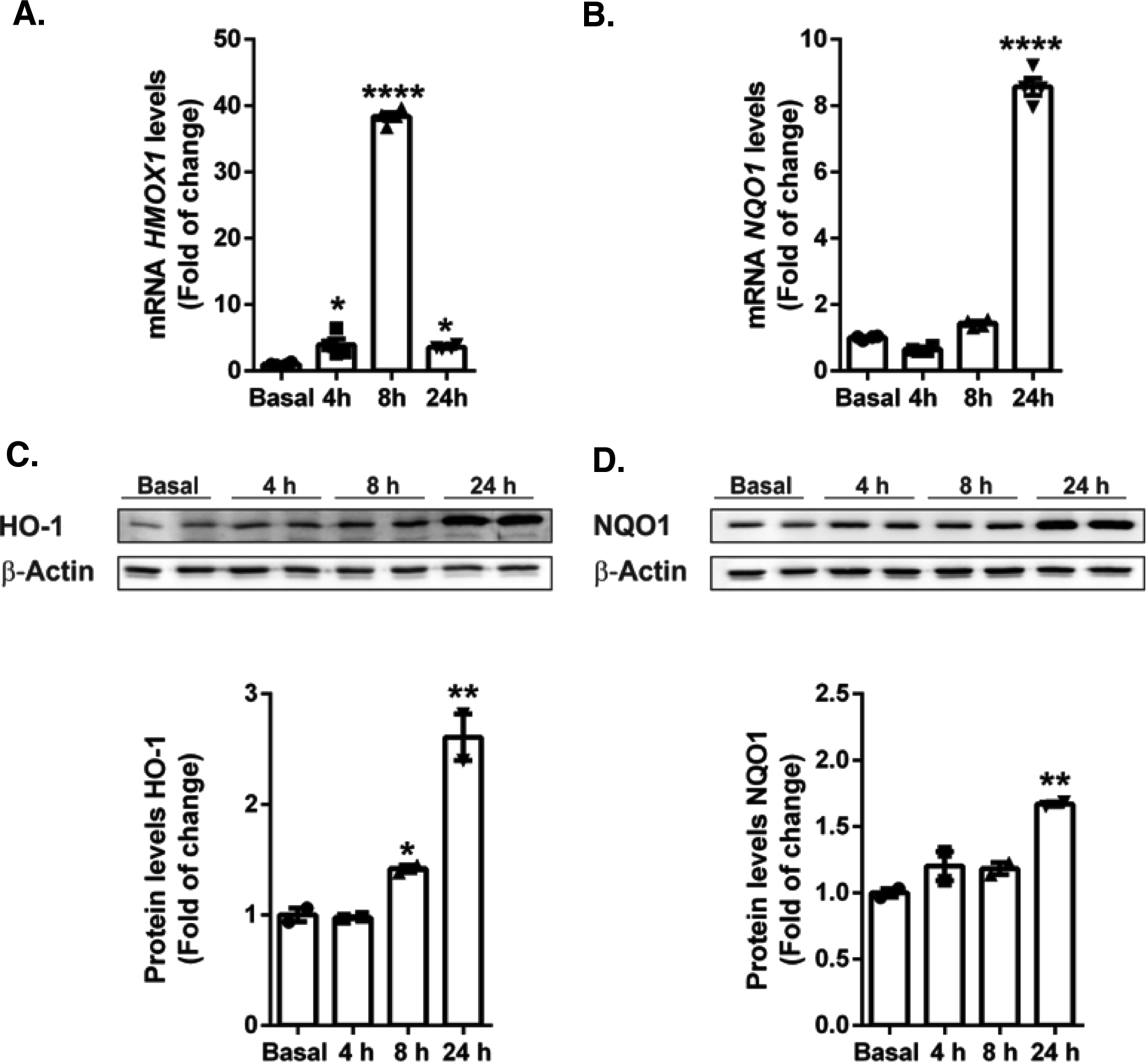
Induction of the NRF2 signature by compound **1d**. SH-SY5Y cells were incubated in the presence of compound **1d** (20 μM) for 4, 8, and 24 h. Quantitative real-time PCR determination of messenger RNA levels of NRF2-regulated genes coding *HMOX1* (A) and *NQO1* (B), normalized by β-actin messenger RNA levels. Dots indicate the mean of *n* = 4 ± S.E.M. (experiments were performed twice). Immunoblot analysis in whole cell lysates of protein levels of HO-1 (C) and NQO1 (D) and β-actin as a loading control. Densitometric quantification of representative blots normalized for β-actin. Experiments were performed in duplicate at least twice. To assess differences between groups, one-way ANOVA followed by a Tukey’s multiple comparison post-test was performed. Asterisks denote statistically significant differences with *p < 0.05 and ****p < 0.0001.

Next, we determined whether compound **1d** could induce the activation of NRF2 target genes. Consistent with the results shown above, compound **1d** increased the mRNA levels of NRF2-dependent genes including heme oxygenase 1 (*HMOX1*) and NAD(*P*)H dehydrogenase quinone 1 (*NQO1*) (Figure 7) in SH-SY5Y cells, in a time-dependent fashion. Moreover, this increase in mRNA levels resulted in an increase in the protein levels of HO-1 and NQO1, respectively, reaching a maximum after 24 h of treatment. The differences in the response times of *HMOX1* and *NQO1* are due to different activation kinetics, as described previously.^51^

All these results confirm that compound **1d** effectively promotes the expression of the antioxidant response enzymes HO-1 and NQO1.

### 2.9 Compound **1d** Induces the NRF2 Transcriptional Signature through KEAP1-Dependent Mechanisms

As mentioned in the Introduction, there are several mechanisms by which the expression of NRF2 can be modulated by small organic molecules. The main control of NRF2 levels is due to its binding to the repressor KEAP1. This protein is a highly reactive redox sensor due to its 27 cysteine (Cys) residues^52^ and therefore could be a target of these small organic molecules.

**Figure 8:**
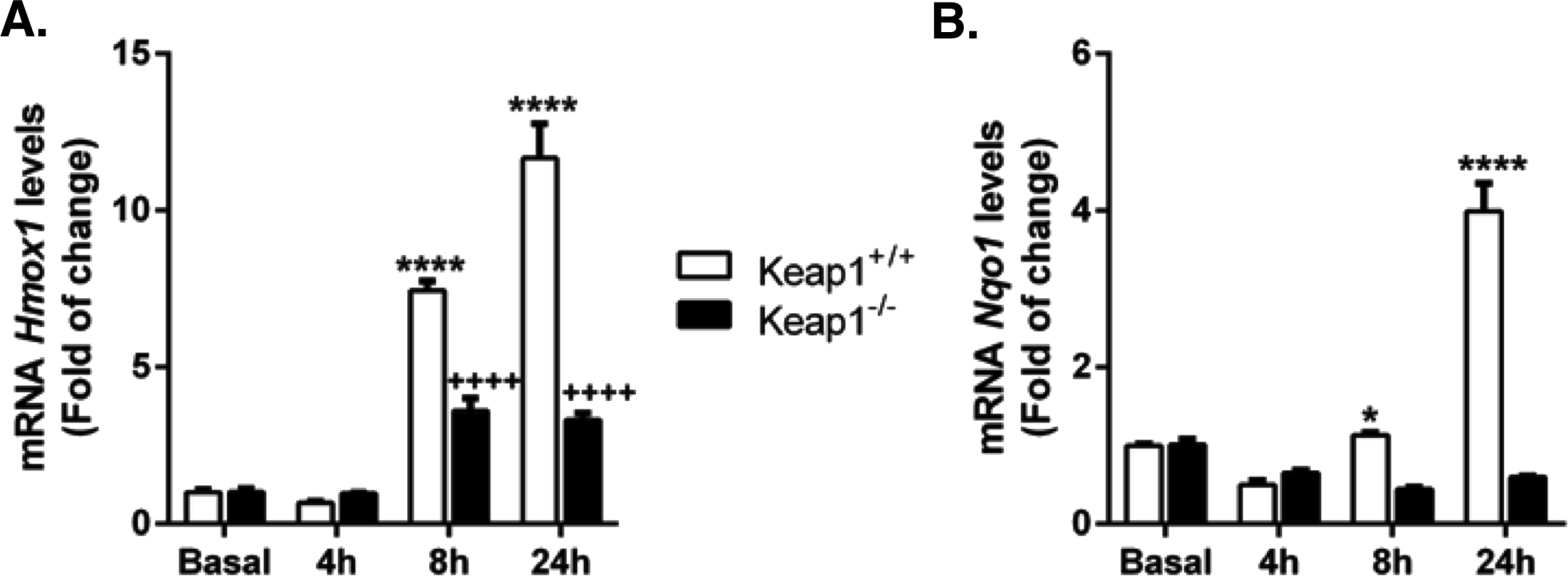
Compound **1d** activates NRF2 signaling through KEAP1-dependent mechanisms. *Keap1*^+/+^ and *Keap1*^–/–^ MEFs were treated with compound **1d** (20 μM) for 4, 8, and 24 h, and mRNA levels for *HMOX1* (A) and *NQO1* (B) were determined by qRT-PCR, normalized to β-actin mRNA levels. Data *n* = 4 ± S.E.M. (experiments were performed twice). Statistical analysis was performed with two-way ANOVA followed by Bonferroni’s post hoc test. *p < 0.05 and ****p < 0.0001 versus *Keap1*^+/+^ MEFs and ^++++^*p* < 0.0001 versus *Keap1*^–/–^ MEFs.

We assessed the implication of KEAP1 on the activation of NRF2 by compound **1d** by using mouse embryonic fibroblasts (MEFs) from wild-type (*Keap1^+/+^*) or KEAP1-deficient (*Keap1^–/–^*) mice. As shown in Figure 8, in *Keap1*^+/+^ cells, compound **1d** increased the mRNA levels of *HMOX1* and *NQO1*, in a time-dependent way. Again, we could observe the different kinetics of both genes. On the contrary, in *Keap1^–/–^* MEFs, compound **1d** is not capable of inducing the expression of *NQO1* and induced the expression of *HMOX1* to a lesser extent than in the wild-type cells. The small increase observed in *HMOX1* mRNA levels may be due to the activation of pathways independent of NRF2 since the *HMOX1* promoter contains binding sites for other transcription factors.^53^ Our results confirm the regulation of NRF2 by compound **1d** in a KEAP1-dependent manner. Although KEAP1-independent mechanisms cannot be completely ruled out, they are probably of less physiological significance.

Regarding the mechanistic details of KEAP1-dependent NFR2 induction, in most of the cases, it depends on Cys alkylation by electrophilic moieties. Alternatively, NRF2 inducers may act by directly interfering with the KEAP1–NRF2 protein–protein interaction.^46^ In order to further investigate the role of KEAP1 in the effect exerted by compound **1d**, we assessed whether our compound could act by releasing NRF2 via inhibition of the protein–protein interaction. Fluorescence polarization and differential scanning fluorimetry assays indicated that compound **1d** is not able to inhibit the NRF2–KEAP1 interaction at 100 μM or lower concentrations (Table 8). Thus, it can be concluded that **1d** interacts with the sensor part of KEAP1. Unlike ferulic and cinnamic acids, **1d** does not contain a side-chain α,β-unsaturated carbonyl moiety, and therefore, the reaction with a Cys residue cannot be due to a Michael addition onto such a functional group. On the other hand, the presence of the catechol structural fragment allows the oxidative generation of a highly electrophilic *ortho*-quinone species, which may bind covalently to the cysteine residues of KEAP1 that act as sensors. Other catechol structures such as dopaminochrome or oxidation products of entacapone, tolcapone, or apomorphine have been described,^54−56^ and they can be easily attacked by thiol groups,^57^ resulting in the release of NRF2 from KEAP1. Similarly, NRF2 induction by epigallocatechin-3-gallate has been explained via the prior oxidation of its catechol moieties to *ortho*-quinones.^58^

**Table 8:** Fluorescence Polarization (FP) Assay and Differential Scanning Fluorimetry (DSF) Assay of the Potential Inhibition of the NRF2–KEAP1 Protein–Protein Interaction by Compound **1d**

compound	FP % inhibition (± S.E.M.)	Δ*T*_i_ (± S.E.M.)^a^
control (10 μM)^b^	94 (± 6)	+19.9 (± 0.2)
**1d** (100 μM)	<10	+0.2 (± 0.1)

a The DSF inflection temperature (*T*_i_) for KEAP1 in the vehicle was 61.3 (± 0.2) °C. Δ*T*_i_ values are differences from the value recorded for KEAP1 alone.

b A naphthalene-2-sulfonamide derivative reported by Jiang et al. (2,2′-(naphthalene-1,4-diylbis(((4-methoxyphenyl)sulfonyl)-azanediyl))diacetic acid) was used as a positive control.^59^

### 2.10 Lymphoblasts from sALS and *SOD1*-ALS Evidence Significant Differences at Basal Levels and after Induction of the NRF2 Signaling Pathway

**Figure 9:**
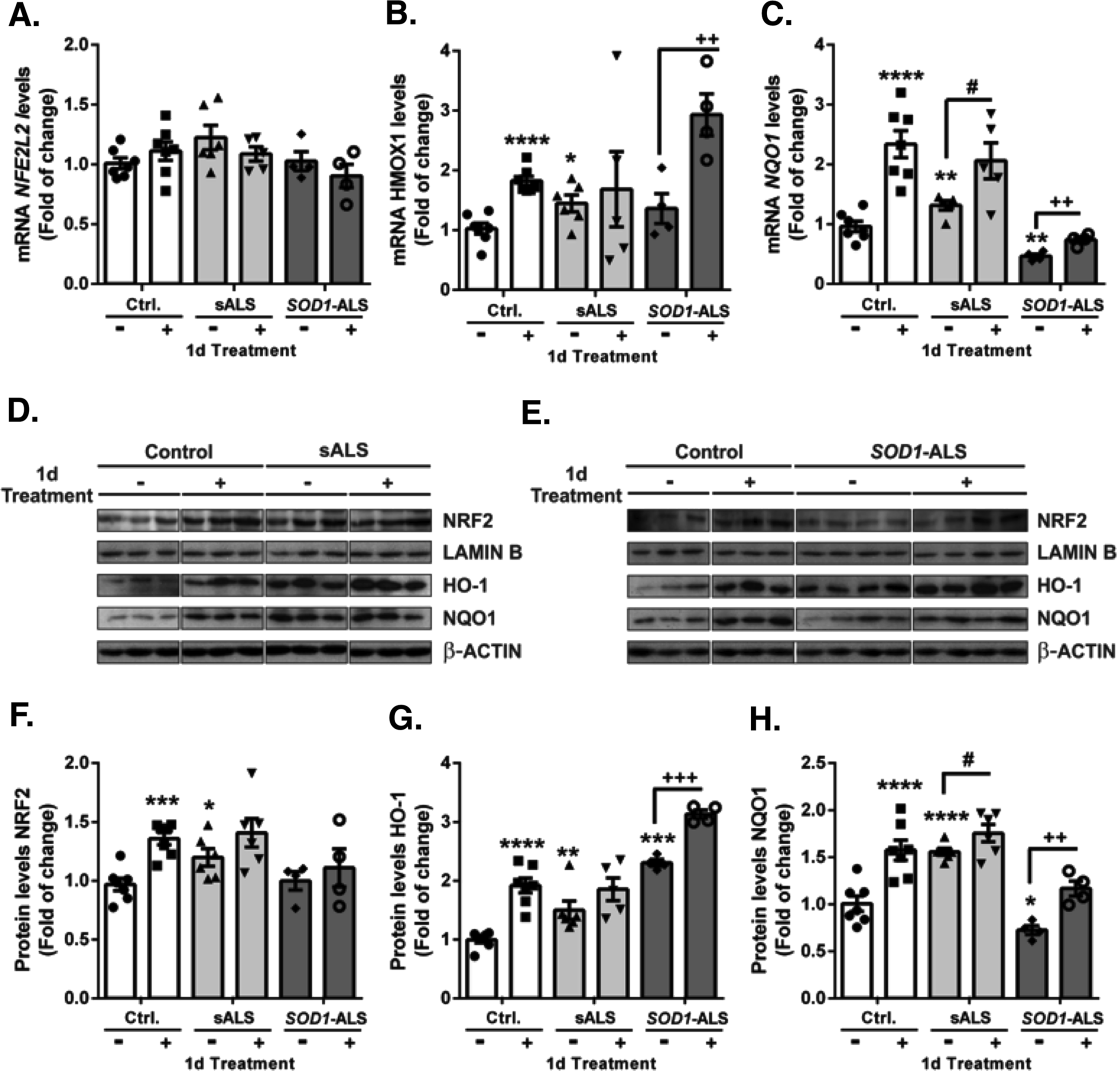
sALS and *SOD1*-mutant ALS lymphoblasts showed significant differences at basal levels and after induction of the NRF2 signaling pathway. Control, sALS, and *SOD1*-ALS lymphoblasts were seeded at an initial density of 1 × 10^6^ cells mL^–1^ in an RPMI medium and synchronized by serum starvation for 12 h. On that point, compound **1d** (20 μM) was added for 24 h. Quantitative real-time PCR determination of messenger RNA levels of *NFE2L2* (A) and NRF2-regulated genes coding *HMOX1* (B) and *NQO1* (C), normalized by β-actin messenger RNA levels. Dots indicate the mean of *n* = 6–7 (controls), *n* = 5–6 (sALS), and *n* = 4 (*SOD1*-ALS) samples ± S.E.M. Immunoblot analysis in the whole control and sALS (D) and *SOD1*-ALS (E) cell lysates of protein levels of NRF2 (F) and Lamin B as a loading control and HO-1 (G), NQO1 (H), and β-actin as a loading control. Densitometric quantification of representative blots normalized for β-actin. Dots indicate the mean of *n* = 6–7 (controls), *n* = 5–6 (sALS), and *n* = 4 (*SOD1*-ALS) samples ± S.E.M. Densitometric quantification of representative blots normalized for Lamin B or β-actin, respectively. Asterisks denote significant differences *p < 0.05, **p < 0.01, ***p < 0.001, and ****p < 0.0001, comparing the indicated groups with the basal condition or the indicated, according to a one-way ANOVA followed by Tukey’s post-test.

Finally, once compound **1d**-dependent induction of NRF2 was proven and characterized, we studied the effect of this compound in a human cell-based model recently developed in our group that mimics ALS.^29^ It is based on immortalized lymphocytes extracted from ALS patients with both familiar ALS associated with a *SOD1* mutation (*SOD1*-ALS) and sporadic ALS (sALS). This human cellular model is an effective platform to study ALS molecular pathology and to evaluate the efficacy of new drugs in a personalized manner.^30^ As previously described,^30^ we corroborated that in sALS lymphoblasts (without treatment), the NRF2 signature was significantly increased in comparison to the control or *SOD1*-ALS lymphoblasts (Figure 9), at the mRNA and protein levels. These data suggested important differences in the molecular mechanisms related to NRF2 signaling linked to the pathology between sALS and *SOD1*-ALS. Therefore, we analyzed the effect of the induction of the NRF2 signaling pathway by compound **1d** (20 μM) in sALS and *SOD1*-ALS lymphoblasts, compared to controls. Treatment of control lymphoblasts with **1d** did not produce changes in *NEF2L2* mRNA levels, as would be expected due to the fact that the NRF2 pathway is mainly regulated at the protein level.^60^ Indeed, the treatment with **1d** significantly increased the levels of the NRF2 protein and consequently the levels of NRF2-dependent genes, *HMOX1* and *NQO1*, both at the mRNA and protein levels (Figure 9). Lymphoblasts from sALS patients have high baseline NRF2 pathway activity compared to control lymphoblasts. Therefore, in lymphoblasts from patients with sALS, treatment with **1d** did not produce a significant increase in NRF2 (compared to untreated sALS lymphoblasts) or the activity of its signaling pathway, either at the mRNA or protein levels. On the contrary, in the lymphoblasts from *SOD1*-ALS patients, where the basal levels of the NRF2 pathway were similar to the levels of the control cells, treatment with **1d** is capable of inducing *HMOX1* and *NQO1* levels in a very significant way, both at the mRNA and protein levels. These results point to a personalized pharmacological strategy for patients with ALS, where modulation of NRF2 should be personalized, based on the molecular alterations displayed by the different types of patients. Our results underline the relevance of NRF2 activators for the treatment of *SOD1*-ALS patients.^30^

## 3 Conclusions

A series of compounds were designed to combine the key structural fragments of the ROCK inhibitor fasudil and the NRF2 inductors/radical scavengers ferulic and caffeic acids and maintain the properties of the parent molecules, in a multitarget strategy. One of the compounds, a fasudil–dihydrocaffeic acid hybrid **1d** was selected for further studies due to its good profile and absence of cytotoxicity and was shown to induce the NRF2 signature by KEAP1-dependent mechanisms. In lymphoblasts obtained from *SOD1*-ALS patients, this compound significantly activated the NRF2 signature, while in sALS, it hardly produced induction, underscoring the potential of this compound in a personalized therapy of ALS, especially for the case of patients with a *SOD1* mutation, and showing the relevance of NRF2 activation as a therapeutic strategy for the treatment of *SOD1*-ALS patients. Thus, compound **1d** can be viewed as an interesting hit on which to base future optimization efforts aimed at improving its activity and ADMET profile.

## 4 Experimental Section

### 4.1 General Experimental Information

All commercial reagents and solvents were used as received. Reactions were monitored by thin-layer chromatography on silica gel-coated aluminum plates containing a fluorescent indicator. Microwave-assisted reactions were performed using a CEM Discover focused microwave reactor. Separations by flash chromatography were performed on conventional silica gel columns or on a Combiflash Teledyne automated flash chromatograph. Melting points were measured with a Kofler-type microscope with a heating plate from Reichert, model 723, and were uncorrected. Infrared spectra were obtained with an Agilent Cary630 FTIR spectrophotometer with a diamond ATR accessory for solid and liquid samples, and wavenumbers are given in cm^–1^. NMR data were obtained using a Bruker Avance spectrometer (CAI de Resonancia Magnética, UCM), working at 250 MHz for ^1^H NMR and 63 MHz for ^13^C NMR; chemical shifts are given in parts per million (δ scale), and coupling constants (*J*) are given in Hertz. Combustion elemental analyses were obtained by the CAI de Microanálisis, Universidad Complutense, using a Leco CHNS-932 combustion microanalyzer. UV–vis measurements were taken with a UV–vis spectrophotometer Cary60 from Agilent, equipped with control and data acquisition Cary WinUV software. The purity of all compounds was >95%, as determined by combustion elemental analysis.

### 4.2 General Procedure for the Synthesis of Isoquinoline-5-sulfonamides **2**

Isoquinoline-5-sulfonic acid (1 mmol), thionyl chloride (1 mL), and a catalytic amount of dimethylformamide (0.01 mmol) were refluxed at 70 °C under an argon atmosphere for 2 h. After this time, the cooled reaction mixture was filtered and washed twice with dichloromethane to afford isoquinoline-5-sulfonyl chloride as a white solid.

A solution of this chloride (1 mmol), pyridine (2 mmol), and triethylamine (1 mmol) in acetonitrile (5 mL) was added dropwise over 20 min to a solution of piperazine or homopiperazine (3 mmol) in acetonitrile (25 mL). During the addition, the temperature was maintained at −5 °C, and then, the reaction was left to warm at room temperature and stirred overnight. The reaction mixture was then concentrated under reduced pressure and redissolved in dichloromethane. The organic phase was then washed with water five times and concentrated under reduced pressure vacuum. The liquid afforded was then purified by chromatography in a silica gel (9:1 dichloromethane:methanol) to afford the pure compounds **2**.

#### 4.2.1 5-(Piperazin-1-ylsulfonyl)isoquinoline (**2a**)

Prepared from isoquinoline-5-sulfonyl chloride (263 mg, 1 mmol), piperazine (258 mg, 3 mmol), pyridine (152 mg, 2 mmol), and trimethylamine (0.14 mL, 1 mmol). Reaction time: 2 h. Yield: 180 mg (65%). Mp: 160–161 °C (lit.,^61^ 162 °C). ^1^H NMR (250 MHz, CDCl_3_): δ 9.35 (d, *J* = 0.9 Hz, 1H), 8.68 (d, *J* = 6.2 Hz, 1H), 8.54 (dt, *J* = 6.2, 0.9 Hz, 1H), 8.37 (dd, *J* = 7.4, 1.3 Hz, 1H), 8.23 (d, *J* = 8.2 Hz, 1H), 7.72 (dd, *J* = 8.1, 7.5 Hz, 1H), 3.18–3.09 (m, 4H), 2.93–2.85 (m, 4H). ^13^CNMR (63 MHz, CDCl_3_): δ 153.7, 145.5, 134.7, 134.3, 132.5, 132.3, 129.5, 126.3, 118.1, 46.8, 45.8.

#### 4.2.2 5-((1,4-Diazepan-1-yl)sulfonyl)isoquinoline **2b** (Fasudil)

Prepared from isoquinoline-5-sulfonyl chloride (263 mg, 1 mmol), homopiperazine (300 mg, 3 mmol), pyridine (152 mg, 2 mmol), and trimethylamine (0.14 mL, 1 mmol). Reaction time: 2 h. Yield: 247 mg (85%), as a viscous oil that solidifies upon standing for 2–3 days to yield a pale-yellow solid. Mp: 128–129 °C. IR (neat): 3292 (NH), 1308 (SO_2_) cm^–1^. ^1^H NMR (250 MHz, CDCl_3_): δ 9.37 (d, *J* = 0.8 Hz, 1H), 8.72 (d, *J* = 6.2 Hz, 1H), 8.47 (d, *J* = 6.2 Hz, 1H), 8.37 (dd, *J* = 7.4, 1.2 Hz, 1H), 8.22 (d, *J* = 8.2 Hz, 1H), 7.76–7.61 (m, 1H), 3.57–3.41 (m, 4H), 2.99 (ddd, *J* = 11.7, 7.8, 4.7 Hz, 4H), 1.90 (s, 1H), 1.85 (dd, *J* = 12.0, 6.0 Hz, 2H). ^13^C NMR (63 MHz, CDCl_3_): δ 153.7, 145.5, 135.0, 133.8, 133.3, 132.0, 129.6, 126.3, 118.0, 51.4, 50.7, 48.0, 47.8, 31.4.

### 4.3 General Procedure for the Synthesis of Hybrid Compounds **1**

The suitable starting material **2** (1 mmol) was dissolved in THF (10 mL) or a 5:1 mixture of dicloromethane and methanol (10 mL). The suitable cinnamic acid derivative (0.9 mmol), 1-ethyl-3-(3-dimethylaminopropyl)carbodiimide (EDCI) (1 mmol), hydrated hydroxybenzotriazole (HOBt·H_2_O) (1 mmol), and diisopropylethylamine or EtN_3_ (2 mmol) were then added to the solution, which was stirred at room temperature until completion, as confirmed by TLC. The reaction mixture was concentrated under reduced pressure and then redissolved in ethanol. The pure compounds (**1**) precipitated at reduced temperature (4 °C) or were purified by silica gel chromatography, using the conditions specified in each case.

#### 4.3.1 3-(3,4-Dihydroxyphenyl)-1-(4-(isoquinolin-5-ylsulfonyl)piperazin-1-yl)prop-2-en-1-one (**1a**)

Prepared from 5-((1,4-piperazin-1-yl)sulfonyl)isoquinoline (**2a**) (277 mg, 1 mmol), caffeic acid (162 mg, 0.9 mmol), EDCI (191 mg, 1 mmol), HOBt (153 mg, 1 mmol), and triethylamine (203 mg, 2 mmol). Reaction time: 72 h. Yield: 78 mg (19%). Mp: 161–162 °C. IR (neat): 3224, 1630 (CO), 1342 (SO_2_) cm^–1^. ^1^H NMR (250 MHz, DMSO): δ 10.29 (s, 1H), 9.50 (d, *J* = 6.1 Hz, 1H), 9.29 (dd, *J* = 11.3, 7.2 Hz, 2H), 9.18 (dd, *J* = 7.4, 1.0 Hz, 1H), 8.68 (t, *J* = 7.8 Hz, 1H), 8.05 (d, *J* = 15.2 Hz, 1H), 7.83 (d, *J* = 1.7 Hz, 1H), 7.79–7.68 (m, 1H), 7.63 (d, *J* = 15.3 Hz, 1H), 7.52 (d, *J* = 8.1 Hz, 1H), 4.57–4.37 (m, 4H), 3.91 (s, 4H). ^13^C NMR (63 MHz, DMSO): δ 165.3, 153.9, 147.8, 145.7, 145.3, 143.1, 134.9, 134.7, 131.4, 131.1, 129.1, 127.0, 126.9, 121.1, 117.4, 115.9, 115.2, 114.1, 31.1 (4C). Elemental analysis calcd. for C_22_H_21_N_3_O_5_S: C, 60.13%; H, 4.82%; N, 9.56%; S, 7.29%. Found: C, 59.87%; H, 5.02%; N, 9.48; S, 7.15.

#### 4.3.2 3-(3,4-Dihydroxyphenyl)-1-(4-(isoquinolin-5-ylsulfonyl)-1,4-diazepan-1-yl)prop-2-en-1-one (**1b**)

Prepared from 5-((1,4-diazepan-1-yl)sulfonyl)isoquinoline (**2b**) (291 mg, 1 mmol), caffeic acid (162 mg, 0.9 mmol), EDCI (191 mg, 1 mmol), HOBt (153 mg, 1 mmol), and triethylamine (203 mg, 2 mmol). Reaction time: 72 h. Yield: 73 mg (18%). Mp: >230 **°**C. IR (neat): 3218, 1638 (CO), 1306 (SO_2_) cm^–1^. ^1^H NMR (250 MHz, DMSO): δ 9.48 (d, *J* = 9.2 Hz, 1H), 8.68 (d, *J* = 4.2 Hz, 1H), 8.44 (t, *J* = 7.1 Hz, 1H), 8.33 (d, *J* = 6.8 Hz, 2H), 7.83 (td, *J* = 7.8, 3.9 Hz, 1H), 7.31 (dd, *J* = 15.2, 6.4 Hz, 1H), 7.09 (s, 1H), 6.99 (d, *J* = 8.0 Hz, 1H), 6.79 (dd, *J* = 14.7, 5.4 Hz, 2H), 3.94–3.48 (m, 8H), 1.91–1.65 (m, 2H). ^13^C NMR (126 MHz, DMSO) (as a mixture of rotamers of the amide): δ 166.4, 166.4, 154.3, 148.3, 148.3, 146.3, 145.8, 145.7, 143.4, 143.2, 134.6, 134.6, 133.2, 133.2, 131.4, 129.7, 127.4, 121.7, 117.8, 116.4, 115.8, 115.7, 114.9, 114.7, 49.9, 49.1, 48.7, 47.9, 47.6, 47.5, 47.0, 45.6, 40.9, 40.8, 40.8, 40.7, 40.6, 40.5, 40.4, 40.4, 40.2, 40.0, 39.9, 30.6, 28.9. Elemental analysis calcd. for C_23_H_23_N_3_O_5_S: C, 60.91%; H, 5.11%; N, 9.27%; S, 7.07%. Found: C, 60.86%; H, 5.07%; N, 9.26%; S, 7.06%.

#### 4.3.3 3-(3,4-Dihydroxyphenyl)-1-(4-(isoquinolin-5-ylsulfonyl)piperazin-1-yl)propan-1-one (**1c**)

Prepared from 5-((1,4-piperazin-1-yl)sulfonyl)isoquinoline (**2a**) (277 mg, 1 mmol), 3-(3,4-dihydroxyphenyl)propanoic acid (163 mg, 0.9 mmol), EDCI (191 mg, 1 mmol), HOBt (153 mg, 1 mmol) and triethylamine (203 mg, 2 mmol). Reaction time: 72 h. Yield: 79 mg (20%). Mp: 158–159 **°**C. IR (neat): 3157, 1599 (CO), 1341 (SO_2_) cm^–1^. ^1^H NMR (250 MHz, MeOD): δ 7.85 (s, 1H), 7.08 (d, *J* = 6.3 Hz, 1H), 7.01 (s, 1H), 6.89 (d, *J* = 8.2 Hz, 1H), 6.84 (d, *J* = 7.5 Hz, 1H), 6.32 (s, 1H), 5.01 (d, *J* = 8.1 Hz, 2H), 4.90 (s, 1H), 2.09–1.99 (m, 2H), 1.91–1.82 (m, 2H), 1.63–1.52 (m, 2H), 1.46–1.36 (m, 2H), 1.13 (t, *J* = 7.3 Hz, 2H), 0.99 (t, *J* = 6.9 Hz, 2H). ^13^C NMR (63 MHz, MeOD): 172.6, 153.3, 145.1, 143.9, 143.6, 134.8, 134.6, 132.2, 132.1, 129.6, 126.7, 119.5, 118.2, 115.4, 115.1, 45.5, 45.4, 41.2, 34.5, 31.0. Elemental analysis calcd. for C_22_H_23_N_3_O_5_S: C, 59.85%; H, 5.25%, N, 9.52%; S, 7.26%. Found: C, 59.60%; H, 5.21%; N, 9.41%; S, (7.25%).

#### 4.3.4 3-(3,4-Dihydroxyphenyl)-1-(4-(isoquinolin-5-ylsulfonyl)-1,4-diazepan-1-yl)propan-1-one (**1d**)

Prepared from 5-((1,4-diazepan-1-yl)sulfonyl)isoquinoline (**2b**) (291 mg, 1 mmol), caffeic acid (162 mg, 0.9 mmol), EDCI (191 mg, 1 mmol), HOBt (153 mg, 1 mmol), and triethylamine (203 mg, 2 mmol). Reaction time: 72 h. Yield: 77 mg (19%). Mp: 194–195 **°**C. IR (neat): 3163, 1613 (CO), 1329 (SO_2_) cm^–1^. ^1^H NMR (300 MHz, DMSO): δ 9.49 (d, *J* = 0.7 Hz, 1H), 8.70 (dd, *J* = 6.2, 4.5 Hz, 2H), 8.60 (d, *J* = 4.1 Hz, 1H), 8.47 (dd, *J* = 7.2, 1.1 Hz, 1H), 8.37–8.19 (m, 2H), 7.93–7.74 (m, 1H), 6.69–6.56 (m, 2H), 6.52–6.37 (m, 1H), 3.66–3.36 (m, 7H), 2.80–2.68 (m, 1H), 2.67–2.55 (m, 2H), 2.47–2.35 (m, 2H), 1.80–1.65 (m, 2H). ^13^C NMR (75 MHz, DMSO) (as a mixture of rotamers of the amide): δ 171.6, 154.0, 145.4, 143.8, 134.2, 132.9, 132.6, 132.5, 131.0, 129.3, 127.1, 119.3, 117.4, 116.3, 115.9, 79.6, 49.3, 49.1, 48.1, 47.8, 46.8, 40.8, 40.6, 40.3, 40.0, 39.7, 39.4, 39.2, 34.8, 34.6, 30.6, 29.6. Elemental analysis calcd. for C_23_H_25_N_3_O_5_S: C, 60.64%; H, 5.53%; N, 9.22%; S, 7.04%. Found: C, 60.39%; H, 5.49%; N, 9.22%; S, 7.02%.

#### 4.3.5 3-(4-Hydroxy-3-methoxyphenyl)-1-(4-(isoquinolin-5-ylsulfonyl)piperazin-1-yl)prop-2-en-1-one (**1e**)

Prepared from 5-((1,4-piperazin-1-yl)sulfonyl)isoquinoline (**2a**) (277 mg, 1 mmol), ferulic acid (174 mg, 0.9 mmol), EDCI (191 mg, 1 mmol), HOBt (153 mg, 1 mmol), and triethylamine (203 mg, 2 mmol). Reaction time: 72 h. Yield: 93 mg (23%). Mp: >230 **°**C. IR (neat): 3003, 1641 (CO) cm^–1^. ^1^H NMR (250 MHz, DMSO): δ 9.49 (s, 1H), 8.71 (d, *J* = 6.2 Hz, 1H), 8.50 (t, *J* = 6.4 Hz, 2H), 8.38 (d, *J* = 7.2 Hz, 1H), 7.90 (d, *J* = 7.8 Hz, 1H), 7.36 (d, *J* = 15.2 Hz, 1H), 7.27 (d, *J* = 1.2 Hz, 1H), 7.11–7.02 (m, 1H), 6.97 (d, *J* = 15.3 Hz, 1H), 6.76 (d, *J* = 8.1 Hz, 1H), 3.81 (s, 3H), 3.79–3.56 (m, 4H), 3.21–3.05 (m, 4H). ^13^C NMR (63 MHz, DMSO): δ 165.0, 153.7, 148.6, 147.9, 145.1, 142.8, 134.6, 134.4, 131.2, 130.8, 128.8, 126.7, 126.6, 122.7, 117.1, 115.5, 114.0, 111.1, 55.8, 45.8 (4C). Elemental analysis calcd. for C_23_H_23_N_3_O_5_S: C, 60.91%; H, 5.11%; N, 9.27%; S, 7.07%. Found: C, 60.46%; H, 5.07%; N, 9.26%; S, 7.09%.

#### 4.3.6 3-(4-Hydroxy-3-methoxyphenyl)-1-(4-(isoquinolin-5-ylsulfonyl)-1,4-diazepan-1-yl)prop-2-en-1-one (**1f**)

Prepared from 5-((1,4-diazepan-1-yl)sulfonyl)isoquinoline (**2b**) (291 mg, 1 mmol), ferulic acid (174 mg, 0.9 mmol), EDCI (191 mg, 1 mmol), HOBt (153 mg, 1 mmol), and triethylamine (203 mg, 2 mmol). Reaction time: 72 h. Yield: 92 mg (22%). Mp: 193–194 **°**C. IR (neat): 3004, 1641 (CO) cm^–1^. ^1^H NMR (500 MHz, DMSO): δ 9.55–9.37 (m, 2H), 8.61 (t, *J* = 5.6 Hz, 1H), 8.36 (dd, *J* = 14.3, 8.2 Hz, 1H), 8.29–8.22 (m, 2H), 7.79–7.71 (m, 1H), 7.33 (dd, *J* = 14.8, 11.9 Hz, 1H), 7.22 (s, 1H), 7.05 (d, *J* = 8.1 Hz, 1H), 6.83 (d, *J* = 15.2 Hz, 1H), 6.76–6.70 (m, 1H), 3.79 (s, 1H), 3.77 (s, 3H), 3.67 (s, 1H), 3.62 (s, 1H), 3.55–3.43 (m, 3H), 3.37 (s, 2H), 1.72 (d, *J* = 23.6 Hz, 2H). ^13^C NMR (75 MHz, DMSO) (as a mixture of rotamers of the amide): δ 166.1, 166.0, 153.9, 149.0, 148.3, 145.4, 145.3, 143.0, 142.8, 134.3, 134.2, 132.9, 132.8, 131.1, 129.3, 127.1, 127.1, 127.0, 122.9, 117.4, 116.0, 114.8, 114.7, 112.1, 56.3, 49.6, 48.2, 47.5, 47.0, 46.7, 45.3, 40.8, 40.6, 40.3, 40.0, 39.7, 39.4, 39.2, 30.3, 28.5. Elemental analysis calcd. for C_24_H_25_N_3_O_5_S: C, 61.66%; H, 5.39%; N, 8.99%; S, 6.86%. Found: C, 61.33%; H, 5.46%; N, 8.80%; S, 6.65%.

#### 4.3.7 3-(4-Hydroxy-3-methoxyphenyl)-1-(4-(isoquinolin-5-ylsulfonyl)piperazin-1-yl)propan-1-one (**1g**)

Prepared from 5-((1,4-piperazin-1-yl)sulfonyl)isoquinoline (**2a**) (277 mg, 1 mmol), 3-(4-hydroxy-3-methoxyphenyl)propanoic acid (176 mg, 0.9 mmol), EDCI (191 mg, 1 mmol), HOBt (153 mg, 1 mmol), and triethylamine (203 mg, 2 mmol). Reaction time: 72 h. Yield: 102 mg (25%). Mp: 156–157 **°**C. IR (neat, cm^–1^): 3210, 1615 (CO), 1343 (SO_2_). ^1^H NMR (250 MHz, MeOD): δ 9.41 (s, 1H), 8.63 (d, *J* = 6.3 Hz, 1H), 8.56 (d, *J* = 6.3 Hz, 1H), 8.46 (d, *J* = 8.2 Hz, 1H), 8.40 (dd, *J* = 7.5, 1.2 Hz, 1H), 7.92–7.83 (m, 1H), 6.70 (d, *J* = 1.4 Hz, 1H), 6.61–6.54 (m, 2H), 3.74 (s, 3H), 3.63–3.55 (m, 2H), 3.48–3.41 (m, 2H), 3.14–3.07 (m, 2H), 2.99–2.92 (m, 2H), 2.74 (t, *J* = 7.0 Hz, 2H), 2.58 (t, *J* = 7.1 Hz, 2H). ^13^C NMR (63 MHz, MeOD): δ 172.5, 153.4, 147.7, 144.8, 143.9, 134.9, 134.7, 132.2, 132.1, 132.1, 129.6, 126.7, 120.7, 118.2, 114.9, 112.0, 55.1, 45.6, 45.5, 45.4, 41.2, 34.5, 31.2. Elemental analysis calcd. for C_23_H_25_N_3_O_5_S: C, 60.64%; H, 5.53%; N, 9.22%; S, 7.04%. Found: C, 60.59%; H, 5.49%; N, 9.22%; S, 7.02%.

#### 4.3.8 3-(4-Hydroxy-3-methoxyphenyl)-1-(4-(isoquinolin-5-ylsulfonyl)-1,4-diazepan-1-yl)propan-1-one (**1h**)

Prepared from 5-((1,4-diazepan-1-yl)sulfonyl)isoquinoline (**2b**) (291 mg, 1 mmol), 3-(4-hydroxy-3-methoxyphenyl)propanoic acid (176 mg, 0.9 mmol), EDCI (191 mg, 1 mmol), HOBt (153 mg, 1 mmol), and triethylamine 203 mg, (2 mmol). Reaction time: 72 h. Yield: 71 mg (17%). Mp: 169–170 **°**C. IR (neat): 3214, 1634 (CO), 1320 cm^–1^. ^1^H NMR (250 MHz, MeOD): δ 9.39 (s, 1H), 8.63 (dd, *J* = 6.2, 2.6 Hz, 1H), 8.49–8.37 (m, 2H), 8.37–8.21 (m, 1H), 7.84 (d, *J* = 2.6 Hz, 1H), 6.78 (s, 1H), 6.65 (t, *J* = 3.7 Hz, 2H), 3.81 (s, 3H), 3.70–3.57 (m, 3H), 3.52 (s, 1H), 3.44 (s, 1H), 3.36 (s, 1H), 3.26 (d, *J* = 5.9 Hz, 2H), 2.88–2.74 (m, 2H), 2.66–2.50 (m, 2H), 1.87 (s, 1H), 1.77 (s, 1H). ^13^C NMR (63 MHz, MeOD) (as a mixture of rotamers of the amide): δ 173.6, 173.6, 153.2, 147.8, 144.8, 143.9, 133.9, 133.8, 133.4, 132.7, 131.7, 129.7, 126.7, 120.9, 118.1, 115.0, 112.2, 55.2, 45.0, 34.9, 34.7, 31.1, 29.3, 27.7, 21.4. Elemental analysis calcd. for C_24_H_27_N_3_O_5_S: C, 61.39%; H, 5.80%; N, 8.95%; S, 6.83%. Found: C, 61.34%; H, 5.74%; N, 8.94%; S, 6.82%.

### 4.4 Docking and Molecular Dynamics

The crystallized ROCK2 human enzyme was obtained from the RCSB Protein Data Bank (PDB ID 4WOT).^39^ Water molecules and cocrystallized ligands were removed. The simplified model was then processed with AutoDockTools (version 1.5.6) to compute the Gasteiger charges and to obtain the AutoDock file. The grid box was determined by calculating the expected center of the interaction area (*x*/*y*/*z* = 43.961/–8.203/103.693), and its size was of *x*/*y*/*z* = 22/26/20 Å. The exhaustiveness was 16, and the number of calculated conformation was 9. Processing of the ligands was performed with UCSF Chimera 1.14. The docking calculation was carried out by AutoDockVina.^62^

Molecular dynamics (MD) simulation was performed using Gromacs 2018.1,^63^ and CHARMM36^64^ was used as a force field. Topologies and parameters of both the ligand and the enzyme were created with CgenFF. The complex was solvated using an SPC water model and then minimized. A two-stage equilibration was performed by applying the NVT ensemble followed by the NPT ensemble for 50,000 steps of 2 fs each. A 10 ns simulation was calculated for each ligand and conformation, with a time step of 2 ps and a cutoff of 1.0 nm. The long-range electrostatic energies were calculated with the PME method, with a fourth-order cubic interpolation and a spaced grid of 0.16 nm. The temperature was regulated at 300 K using a Berendsen thermostat with a coupling constant of 0.1 ps. The pressure was fixed at 1 bar and controlled with a Parrinello–Rahman barostat with a coupling constant of 2 ps, and a compressibility of 4.5 × 10^–5^ bar^–1^ was employed.

Root-mean-square displacement (RMSD) between any snapshot and the minimized state of the system was calculated to evaluate the equilibrium of the system during simulation. The mobility of the ligand around the enzyme was evaluated by measuring distances between atoms present in residues from the hinge region of the enzyme and different atoms present in the ligands. An estimation of the binding energy was also calculated by using the molecular mechanics Poisson–Boltzmann surface area (MM-PBSA) method using the last 200 MD snapshots (2 ns) from each simulation.

### 4.5 Serum Albumin Binding

Human serum albumin (HAS) was diluted in phosphate buffer solution (pH = 7.2, 50 mM, [NaCl] = 150 mM) to a final concentration of 2 μM. Then, compound **1d** was sequentially added to achieve increasing concentrations (0, 0.2, 0.8, 1.4, 2, 8, 14, and 20 μM). After each addition, the mixture was incubated at rt for 5 min, and then, the emission fluorescence spectra of HAS (λ = 280 nm) were measured using a Fluorometer Max-4P (Horiba Jobin Yvon). The data obtained were further processed using the Stern–Volmer and Scatchard models in GraphPad Prism 8.0 software.

### 4.6 Liver Microsome Stability Assay

Human liver microsomes and reduced nicotinamide adenine dinucleotide phosphate (NADPH) were purchased from Fisher Scientific SL. This assay provides information on the metabolic stability of early drug discovery compounds based on liver microsomes. Microsome stability was tested by incubating 8 μM of the test compound and verapamil (as control) with 1.0 mg/mL hepatic microsomes (pooled human liver microsomes) in 0.1 M potassium phosphate buffer (pH 7.4) with 5 mM MgCl_2_. The reaction was initiated by adding NADPH (1 mM final concentration). Aliquots of 150 μL were collected at defined time points (0, 5, 15, 30, and 60 min) and added to cold acetonitrile (150 μL) containing an internal standard (5 μg/mL warfarin) to stop the reaction and precipitate the protein. After stopping the reaction, the samples were centrifuged at 4 °C for 15 min, and the loss of the parent compound was analyzed by high-pressure liquid chromatography coupled to mass spectrometry (HPLC-MS). Data were log transformed and represented as half-life. All experiments were conducted in duplicates.

### 4.7 Antioxidant Activity Test

The antioxidant activity was determined by the 2,2-diphenyl-1-picrylhydrazyl hydrate (DPPH) assay.^65−67^ All the measurements were performed in an 80% MeOH solution, with the final reaction volume being 3 mL. A stock of DPPH solution in DMSO (10^–2^ M) was prepared and stored at −20 °C, and the same procedure was performed with the antioxidant solution. Then, the DPPH stock solution was diluted to 2 mL with a final concentration of 150 μM (final volume of 3 mL and final concentration of 100 μM). The antioxidant stock was sequentially diluted to 1 mL at 3, 9, 30, 90, and 300 μM (final volume of 3 mL and final concentrations of 1, 3, 10, 30, and 100 μM). The mixture was incubated at room temperature for 30 min. Once this time had passed, the absorbance was measured in a spectrophotometer at 517 nm. Trolox, caffeic acid, and ferulic acid were used as a standard (1, 3, 10, 30, and 100 μM final concentrations). A blank using 80% MeOH solution instead of the corresponding antioxidant was used in each assay. Two independent measurements of each sample were performed. Data were processed using Origin software, and sigmoidal fitting according to the DoseResp function was performed to extrapolate the IC_50_ (μM) of each antioxidant.

### 4.8 ROCK Inhibition Studies

ROCK-II (ROKa) (5–20 mU diluted in 50 mM Tris pH 7.5, 0.1 mM EGTA, 0.1% 2-mercaptoethanol, and 1 mg/mL BSA) was assayed against the Long S6 substrate peptide (KEAKEKRQEQIAKRRRLSSLRASTSKSGGSQK) in a final volume of 25.5 μL containing 50 mM Tris pH 7.5, 0.1 mM EGTA, 30 μM Long S6 substrate peptide, 10 mM magnesium acetate, and 0.02 mM [33P-*g*-ATP] (50–1000 cpm/pmol) and incubated for 30 min at room temperature. Assays were stopped by addition of 5 μL of 0.5 M (3%) orthophosphoric acid and then harvested onto P81 Unifilter plates with a wash buffer of 50 mM orthophosphoric acid.

### 4.9 Cell Culture and Reagents

Human embryonic kidney (HEK) 293T cells were grown in Dulbecco’s modified Eagle’s medium (DMEM) supplemented with 10% fetal bovine serum and 80 μg/mL gentamycin. Transient transfections were performed with calcium phosphate using reagents from Sigma. The *Keap1*^–/–^ mouse embryo fibroblasts (MEFs) and their corresponding wild-type *Keap1*^+/+^ MEFs were kindly provided by Dr. Ken Itoh (Department of Stress Response Science, Center for Advanced Medical Science, Hirosaki University, Japan). MEFs were grown in DMEM supplemented with 10% fetal bovine serum, 1% penicillin/streptomycin, and 2 mm l-glutamine. SH-SY5Y cells were grown in DMEM and Ham’s F12 supplemented with 10% fetal bovine serum (FBS) and 1% penicillin/streptomycin. The medium was changed to serum-free DMEM without antibiotics 16 h before treatments. DMF (cat. no. 242926, Sigma-Aldrich) was used at 20 μM for 16 h.

### 4.10 Lymphoblastic Cell Lines

Peripheral blood samples of all the individuals enrolled in this study were collected after written informed consent of the patients or their relatives (demographic information is presented in Table 9) to establish the lymphoblastoid cell lines (LCLs), by infecting peripheral blood lymphocytes with the Epstein–Barr virus (EBV), as previously described.^68^ Participants or their relatives gave written informed consent. This study was approved by the Hospital Doce de Octubre and the Spanish Council of Higher Research Institutional Review Boards. All patients were diagnosed by applying the revised El Escorial criteria.^69^ Control healthy individuals were recruited separately and did not have any known neurological disorder. Genetic testing for *SOD1*, TARDBP, FUS, and C9ORF72 was performed in all cases.

**Table 9:** Demographic and Clinical Characterization of Subjects Included in This Study^a^

	control (*n* = 7)	sALS (*n* = 6)	*SOD1*-ALS (*n* = 4)
Gender (M/F)	5/2	3/3	4/0
Family history	no	no	yes
Age range			
at sampling	52–75	55–76	46–54
Site of onset (*N*)			
bulbar	NA	4	
spinal	NA	1	4
respiratory	NA	1	
Mutation (*n*)			
*SOD1* het N65S *SOD1*			1
*SOD1* het p.Leu117Val			2
*SOD1* het p.ASn139His			1

a M, male; F, female; NA, not applicable.

LCLs were grown in suspensions in T flasks, in an RPMI-1640 medium containing 2 mM l-glutamine, 100 μg/mL streptomycin/penicillin, and 10% (v/v) fetal bovine serum (FBS) and maintained in a humidified 5% CO_2_ incubator at 37 °C.

### 4.11 MTT Viability Assay

HEK293T cells were plated in 24-well culture plates (75,000 cells/well) and incubated in a CO_2_ incubator. The next day, treatment was given according to the experimental requirement. Sixteen hours later, 50 μL of MTT solutions from the stock (5 mg/mL) was added, and cells were incubated in a CO_2_ incubator in the dark for 2 h. The medium was removed, and formazan crystals formed by the cells were dissolved using 500 μL of DMSO followed by transfer in 96-well plates. The absorbance was read at a 570 nm wavelength on a multiwell plate reader.^70^

### 4.12 Plasmids and Luciferase Assay

Transient transfections of HEK293T cells were performed with the expression vectors for TK-Renilla (Promega, Madison, CA) and the expression vector ARE-LUC (Dr. J. Alam, Dept. of Molecular Genetics, Ochsner Clinic Foundation, New Orleans, LA). Cells were seeded on 24-well plates (75,000 cells per well), cultured for 16 h, and transfected using calcium phosphate. Eight hours after transfections, cells were treated with 3 different concentrations of the compounds (6, 20, and 60 μM). DMF (20 μM) was used as a positive control. After 16 h, the cells were lysed and assayed with a dual-luciferase assay system (Promega) according to the manufacturer’s instructions. Relative light units were measured in a GloMax 96 microplate luminometer with dual injectors (Promega).

### 4.13 Preparation of Nuclear and Cytosolic Extracts

SH-SY5Y cells were seeded in p100 plates (1 × 10^6^ cells/plate) and treated with 20 μM compound **1d**. Cytosolic and nuclear fractions were prepared as described previously.^71^ Briefly, cells were washed with cold PBS and harvested by centrifugation at 1100 rpm for 10 min. The cell pellet was resuspended in 3 pellet volumes of cold buffer A (20 mm HEPES, pH 7.0, 0.15 mm EDTA, 0.015 mm EGTA, 10 mm KCl, 1% Nonidet P-40, 1 mm phenylmethylsulfonyl fluoride, 20 mm NaF, 1 mm sodium pyrophosphate, 1 mm sodium orthovanadate, and 1 μg/mL leupeptin) and incubated in ice for 30 min. Then, the homogenate was centrifuged at 500*g* for 5 min. The supernatants were taken as the cytosolic fraction. The nuclear pellet was resuspended in 5 volumes of cold buffer B (10 mm HEPES, pH 8.0, 0.1 mm EDTA, 0.1 mm NaCl, 25% glycerol, 1 mm phenylmethylsulfonyl fluoride, 20 mm NaF, 1 mm sodium pyrophosphate, 1 mm sodium orthovanadate, and 1 μg/mL leupeptin). After centrifugation in the same conditions indicated above, the nuclei were resuspended in loading buffer containing 0.5% SDS. The cytosolic and nuclear fractions were resolved in SDS-PAGE and immunoblotted with the antibodies indicated in Supporting Information Table S4.

### 4.14 Immunoblotting

Whole cell lysates were prepared in RIPA-buffer (25 mM Tris-HCl, pH 7.6, 150 mM NaCl, 1 mM EGTA, 1% Igepal, 1% sodium deoxycholate, 0.1% SDS, 1 mM PSMF, 1 mM Na_3_VO_4_, 1 mM NaF, 1 μg/mL aprotinin, 1 μg/mL leupeptin, and 1 μg/mL pepstatin). Whole cell lysates, cytosolic and nuclear fractions containing 25 μg of whole proteins from SH-SY5Y, or lymphoblast-treated cells were loaded for SDS-PAGE electrophoresis. Immunoblots were analyzed as described previously.^48^ The primary antibodies used are described in Supporting Information Table S4.

### 4.15 Analysis of mRNA Levels by Quantitative Real-Time PCR

Total RNA extraction, reverse transcription, and quantitative polymerase chain reaction (PCR) were done as detailed in previous articles.^72^ Primer sequences are shown in Supporting Information Table S5. Data analysis was based on the ΔΔCT method with normalization of the raw data to housekeeping genes (Applied Biosystems). All PCRs were performed in triplicates.

### 4.16 Statistical Analyses

Data are presented as means ± SEM. To determine the statistical test to be used, we employed GraphPad Instat 3.1, which includes the analysis of the data to normal distribution via the Kolmogorov–Smirnov test. In addition, statistical assessments of differences between groups were analyzed (GraphPad Prism 6, San Diego, CA) by unpaired Student’s *t*-tests when normal distribution and equal variances were fulfilled or by the nonparametric Mann–Whitney test. One- and two-way ANOVA with the post hoc Newman–Keuls test or Bonferroni’s test was used, as appropriate.

## Supporting Information Available

Complementary experimental data and copies of spectra (PDF)
PDB files for fasudil and compound **1d** docked in ROCK2 (PDB) (PDB)
Molecular formula strings (CSV)

## Supplementary Material

jm1c01255_si_001.pdf

jm1c01255_si_002.pdb

jm1c01255_si_003.pdb

## Abbreviations

ADME: absorption, distribution, metabolism, and excretion
AKT: protein kinase 1
ALS: amyotrophic lateral sclerosis
ANOVA: analysis of variance
ARE: antioxidant response element
C9ORF72: chromosome 9 open reading frame 72
CUL3/RBX1: cullin 3 and RING-box protein1
DMF: dimethyl fumarate
DPPH: 2,2-diphenyl-1-picrylhydrazyl hydrate
EDCI: 1-ethyl-3-(3-dimethylaminopropyl)carbodiimide
ERK: extracellular signal-regulated kinase
fALS: familial amyotrophic lateral sclerosis
FDA: food and drug administration
FUS: fused in sarcoma (protein)
GAPDH: glyceraldehyde 3-phosphate dehydrogenase
GSK-3: glycogen synthase kinase 3
*HMOX1*: heme oxygenase 1
HOBt: 1-hydroxybenzotriazole
JNK: c-Jun N-terminal kinase
KEAP1: cap’n’collar homology (ECH)-associated protein 1
LUC: luciferase
MAPK: mitogen-activated protein kinase
MEFs: mouse embryonic fibroblasts
MM-PBSA: molecular mechanics Poisson–Boltzmann surface area
MNs: motoneurons
MTDL: multitarget-directed ligand
MTT: 3-(4,5-dimethylthiazol-2-yl)-2,5-diphenyltetrazolium bromide
NADPH: nicotinamide adenine dinucleotide phosphate, reduced form
NQO1: NAD(*P*)H dehydrogenase quinone 1
NRF2: nuclear factor erythroid 2-related factor 2
PAINS: pan-assay interference compounds
ROCK: Rho-associated protein kinase
sALS: sporadic amyotrophic lateral sclerosis
*SOD1*: superoxide dismutase 1
TDP-43: TAR DNA-binding protein 43
TLC: thin-layer chromatography
β-TrCP: β-transducin repeat-containing protein
