# Supplementary material for: Multitarget Hybrid Fasudil Derivatives as a New Approach to the Potential Treatment of Amyotrophic Lateral Sclerosis
Source: J Med Chem. 2022 Jan 5;65(3):1867–82. doi: 10.1021/acs.jmedchem.1c01255 (PMC9132363; doi:10.1021/acs.jmedchem.1c01255)
Supplement: Supplementary file 1 — jm1c01255_si_001.pdf [file jm1c01255_si_001.pdf]

Supporting Information

Multitarget hybrid fasudil derivatives as a new approach to the potential treatment of Amyotrophic Lateral Sclerosis

Olmo Martín-Cámara[a‡], Marina Arribas[b‡], Geoffrey Wells[c], Marcos Morales-Tenorio[d], Ángeles Martín-Requero[d,e], Gracia Porras[d] Ana Martínez[d,e], Giorgio Giorgi[a], Pilar López-Alvarado[a], Isabel Lastres-Becker[b,e,*], J. Carlos Menéndez[a,*]

[a] Unidad de Química Orgánica y Farmacéutica, Departamento de Química en Ciencias Farmacéuticas, Facultad de Farmacia, Universidad Complutense, 28040 Madrid, Spain.

[b] Instituto de Investigaciones Biomédicas "Alberto Sols" UAM-CSIC, Arturo Duperier,4 Madrid, Spain. Department of Biochemistry, School of Medicine, Universidad Autónoma de Madrid, Spain. Institute Teófilo Hernando for Drug Discovery, Universidad Autónoma de Madrid, 28029 Madrid, Spain.

[c] Department of Pharmaceutical and Biological Chemistry, UCL School of Pharmacy. University College London, 29/39 Brunswick Square, London WC1N 1AX (UK).

[d] Centro de Investigaciones Biológicas Margarita Salas, CSIC. Ramiro de Maeztu 9, 28040, Madrid, Spain.

[e] Centro de Investigación Biomédica en Red de Enfermedades Neurodegenerativas (CIBERNED), Instituto de Salud Carlos III, 28031 Madrid (Spain).

‡ These authors contributed equally.

Correspondence: ilbecker@iib.uam.es (I.L.-B.); josecm@farm.ucm.es (J.C.M).

Table of contents

Table S1: Computational drug-likeness study of compounds **1** using SwissADME S2
Table S2: Additional ADMET predictions obtained with ADMElab 2.0 S2
Table S3. Response to the luciferase assay for three different concentrations of compounds **1** S3
Table S4. List of antibodies used in this study S3
Table S5. List of primers used in this study S3
Figure S1. Lack of effect of compound **1d** against aberrant TDP-43 phosphorylation in sporadic ALS and control lymphoblasts S4
Copies of spectra S5
Copies of representative HPLC chromatograms S13
References S15

Table S1. Computational drug-likeness study of compounds **1** using Swiss ADME

Cmpd.	Rotatable bonds	TPSA[a]	logP o/w[b]	log S[c]	GI absorption[1]	Lipinski violations[2]	PAINS alerts[3]
**1a**	5	119.42	1.73	-3.81	High	0	1
**1b**	5	119.42	1.97	-4.11	High	0	1
**1c**	6	119.42	1.62	-3.63	High	0	1
**1d**	6	119.42	1.90	-3.94	High	0	1
**1e**	6	108.42	2.14	-4.02	High	0	0
**1f**	9	108.42	2.30	-4.33	High	0	0
**1g**	9	108.42	2.17	-3.85	High	0	0
**1h**	7	108.42	2.38	-4.15	High	0	0

[a] Topological Polar Surface Area calculated according to ref. 4. [b] Consensus Log P o/w average of 5 prediction methods. [c] ESOL topological method implemented from ref. 5.

Table S2. Additional ADMET predictions obtained with ADMElab 2.0

Compound	HIA[a]	Caco-2[b]	hERG[c]
**1a**	0.135	-5.985	0.605
**1b**	0.096	-6.125	0.671
**1c**	0.974	-6.182	0.647
**1d**	0.942	-6.247	0.715
**1e**	0.048	-5.460	0.737
**1f**	0.044	-5.688	0.814
**1g**	0.557	-5.853	0.843
**1h**	0.162	-5.970	0.902

[a] Human intestinal absorption (HIA); the value represents the possibility of being HIA+, HIA+ (< 30 %), HIA- (> 30%). [b] Caco-2 permeability predicted value (Caco-2); this parameter is optimal when is higher than -5.15 Log unit. [c] Ether-a-go-go Related-Gene (hERG) probability of being a blocker.

Table S3. Response to the luciferase assay for three different concentrations of compounds **1** (6, 20 and 60 μM). Dimethyl fumarate (DMF) and sulforaphane (SFN) were used as positive controls.

Compound	MTT (%) (20 μM)	Luciferase assay		
		6 μM	20 μM	60 μM
**Fasudil**	< 20%	1.3 ± 0.21	1.0 ± 0.06	2.9 ± 0.49
**1a**	< 20%	0.7 ± 0.06	0.8 ± 0.02	1.2 ± 0.08
**1b**	< 20%	0,5 ± 0.06	3.1 ± 0.04	4.1 ± 0.06
**1c**	< 20%	1.7 ± 0.09	2.2 ± 0.09	5.8 ± 0.23
**1d**	< 20%	2.5 ± 0.05	4.4 ± 0.27	13.6 ± 1.60
**1e**	< 20%	1.2 ± 0.11	1.1 ± 0.16	1.2 ± 0.14
**1f**	< 20%	0.7 ± 0.06	0.8 ± 0.02	1.2 ± 0.08
**1g**	< 20%	1.0 ± 0.01	0.8 ± 0.04	1.5 ± 0.22
**1h**	< 20%	1.2 ± 0.09	0.9 ± 0.02	1.2 ± 0.03
**DMF**			4.9 ± 0.35	
**SFN**		4.0 ± 0.08		

Table S4. List of antibodies used in this study.

Antibody	Source	Catalog number	Dilution
β-ACTIN	Santa Cruz Biotechnologies	sc-1616	1:5000
HO-1	Chemicon International	AB1284	1:1000
p-AKT	Cell Signaling	#4058	1:1000
AKT	Santa Cruz Biotechnologies	sc-1618	1:1000
pERK1/2	Cell Signaling	#9106	1:1000
ERK1/2	Cell Signaling	#4695	1:1000
LAMIN B	Santa Cruz Biotechnologies	sc-6217	1:1000
NRF2	Abyntek	AJ1555a	1:2000
NQO1	Abcam	ab2346	1:2000
p-p38	Cell Signaling	#9211	1:1000
p38	Cell Signaling	#9212	1:1000
pSAPK/JNK	Cell Signaling	#9251	1:1000
SAPK/JNK	Cell Signaling	#9252	1:1000

Table S5. List of primers used in this study.

Gene product	Forward primer	Reverse primer
β-ACTIN	5′ TCCTTCCTGGGCATGGAG 3′	5′ AGGAGGAGCAATGATCTTGATCTT 3′
HMOX1	5′ TGCTCAACATCCAGCTCTTTGA 3′	5′ GCAGAATCTTGCACTTTGTTGCT 3′
NFE2L2	5′CCCGAAGCACGCTGAAGGCA 3′	5′ CCAGGCGGTGGGTCTCCGTA 3′
NQO1	5′ GTTCATAGGAGAGTTTGCTT 3′	5′ TAGAACCTCAACTGACACTT 3′
TBP	5′ TGCACAGGAGCCAAGAGTGAA 3′	5′ CACATCACAGCTCCCCACCA 3′
TXN	5′ TTTCAGGAAGCCTTGGACGCT 3′	5′ GCAACATCCTGACAGTCATCCAC 3′

Figure S1. Lack of effect of compound **1d** against aberrant TDP-43 phosphorylation in sporadic ALS and control lymphoblasts

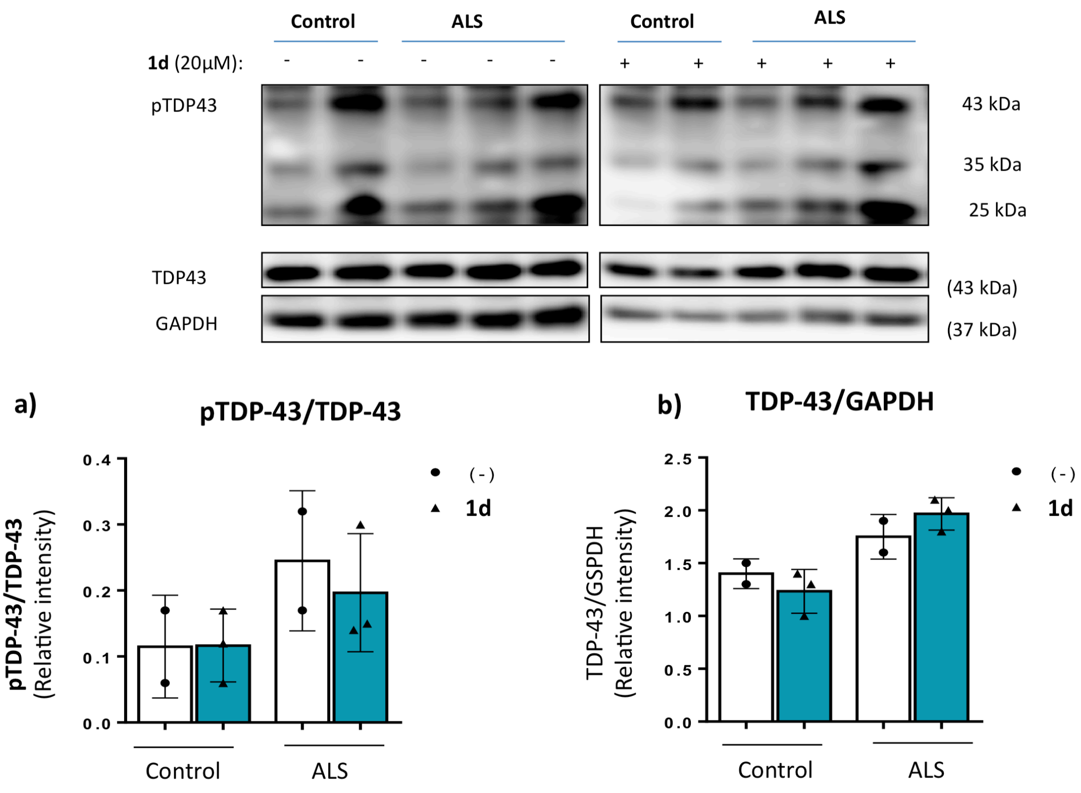

Representative immunoblot showing the phosphorylation status of TDP-43 in immortalized lymphocytes from control and sporadic ALS individuals. Cells were seeded at an initial density of 1 x 10^6 x ml^{-1} in the absence or presence of compound **1d** (20 µM). 24 hours after compound addition, cells were harvested and processed for Western blotting analysis. The plot in the left panel (a) represents the quantification of the bands of 43 KD of pTDP-43 normalized by total-TDP-43. The densitometric analyses represent the mean ± SEM of different observations carried out in 2 control and 3 ALS patients.

Conclusion: Compound **1d** does not reduce aberrant TDP-43 phosphorylation (a) neither TDP-43 expression (b) in lymphoblasts from ALS patients.

Copies of spectra

(*E*)-3-(3,4-dihydroxyphenyl)-1-(4-(isoquinolin-5-ylsulfonyl)piperazin-1-yl)prop-2-en-1-one (1a)

^{1}H-RMN

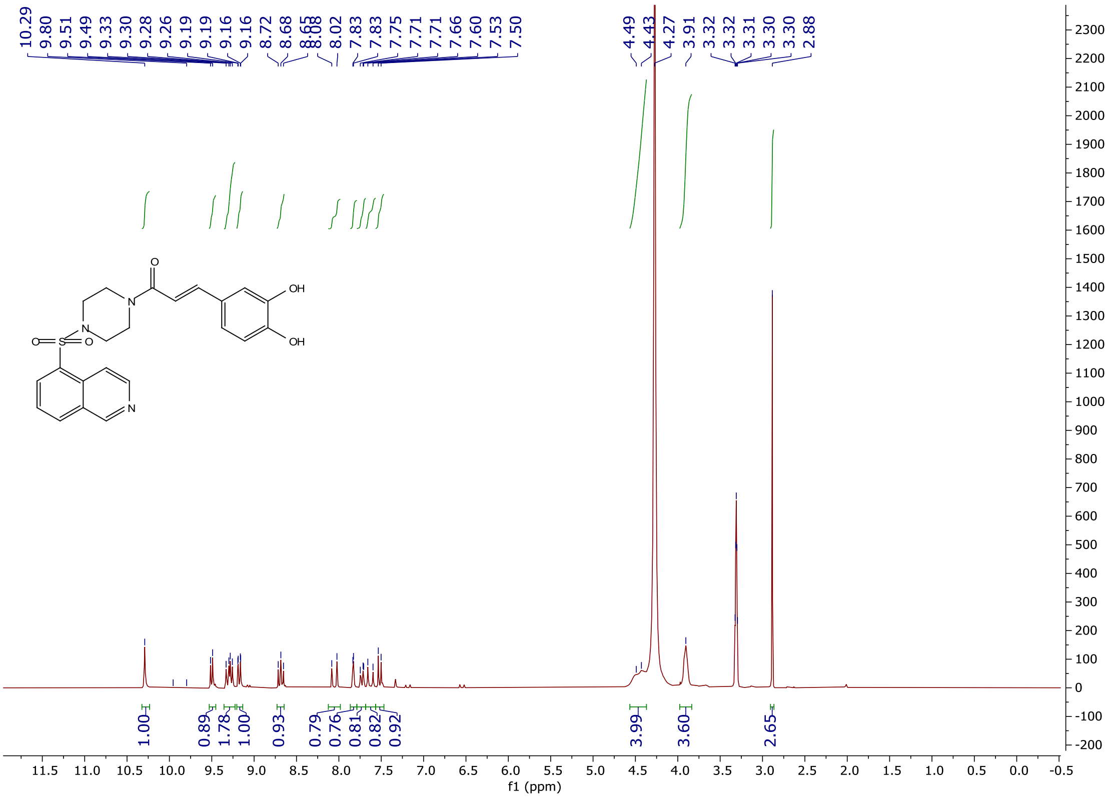

^{13}C-RMN

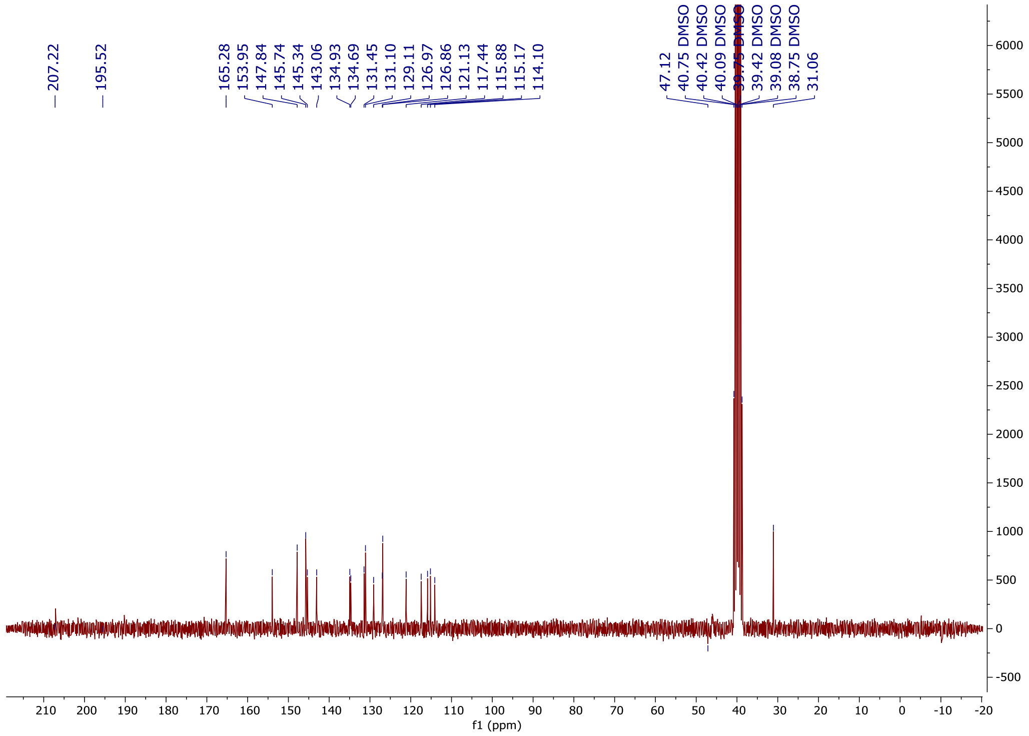

(*E*)-3-(3,4-dihydroxyphenyl)-1-(4-(isoquinolin-5-ylsulfonyl)-1,4-diazepan-1-yl)prop-2-en-1-one (1b)

^{1}H-RMN

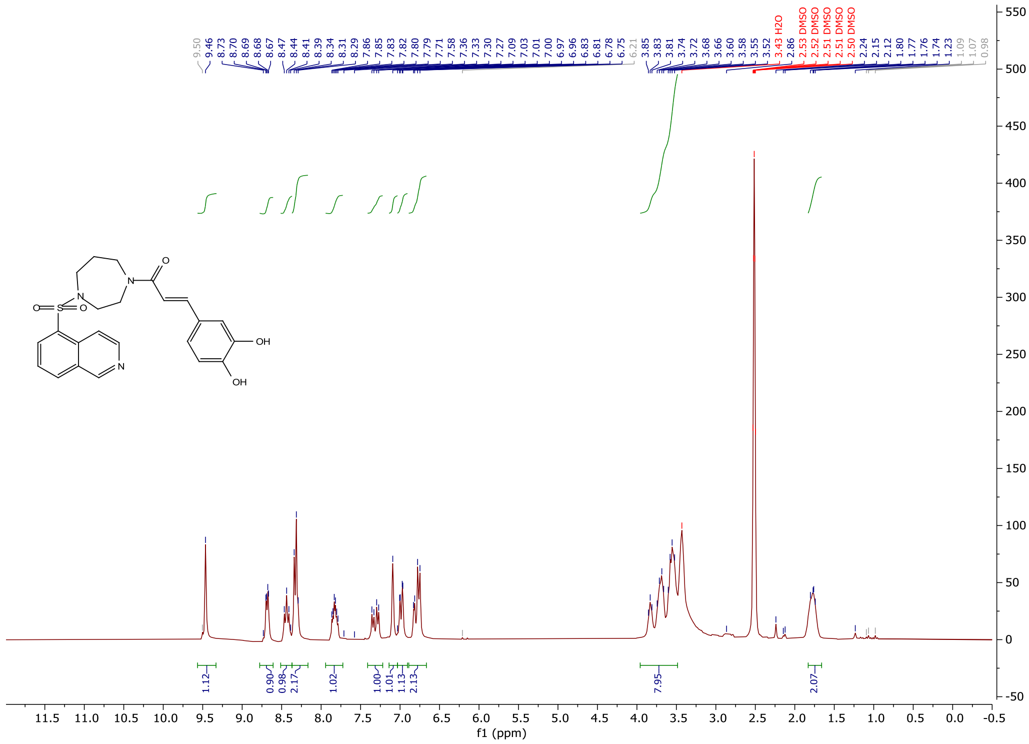

^{13}C-RMN

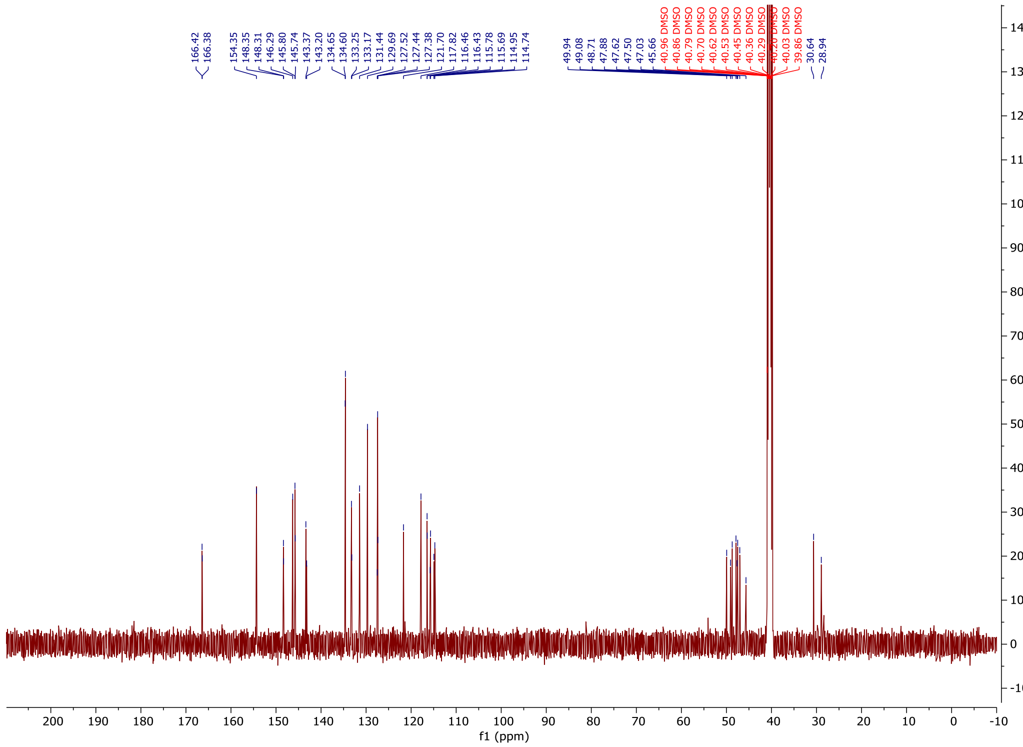

3-(3,4-Dihydroxyphenyl)-1-(4-(isoquinolin-5-ylsulfonyl)piperazin-1-yl)propan-1-one (1c)

^{1}H-RMN

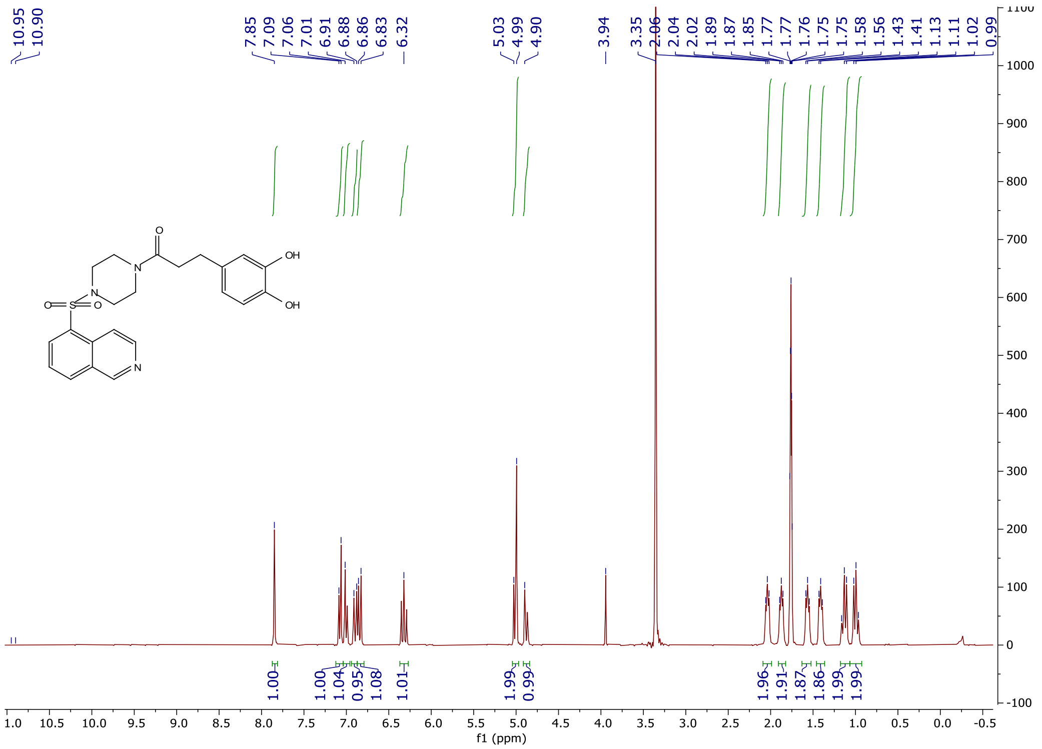

^{13}C-RMN

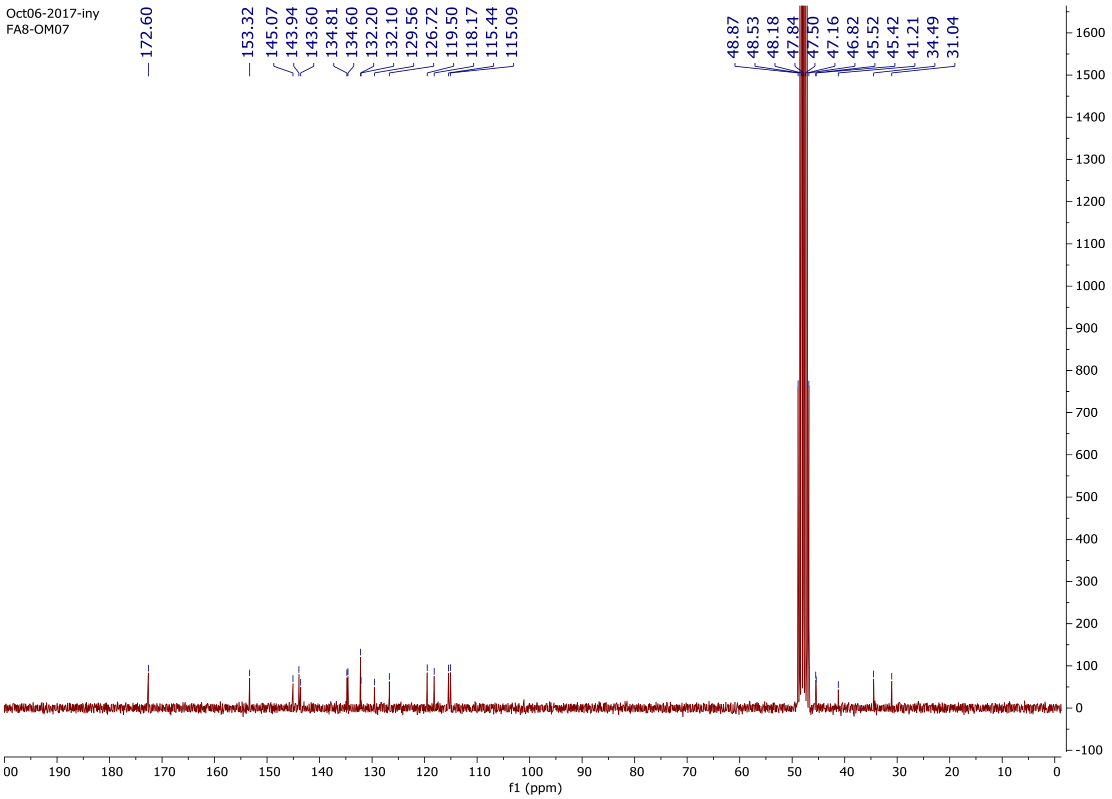

3-(3,4-Dihydroxyphenyl)-1-(4-(isoquinolin-5-ylsulfonyl)-1,4-diazepan-1-yl)propan-1-one (1d)

^{1}H-RMN

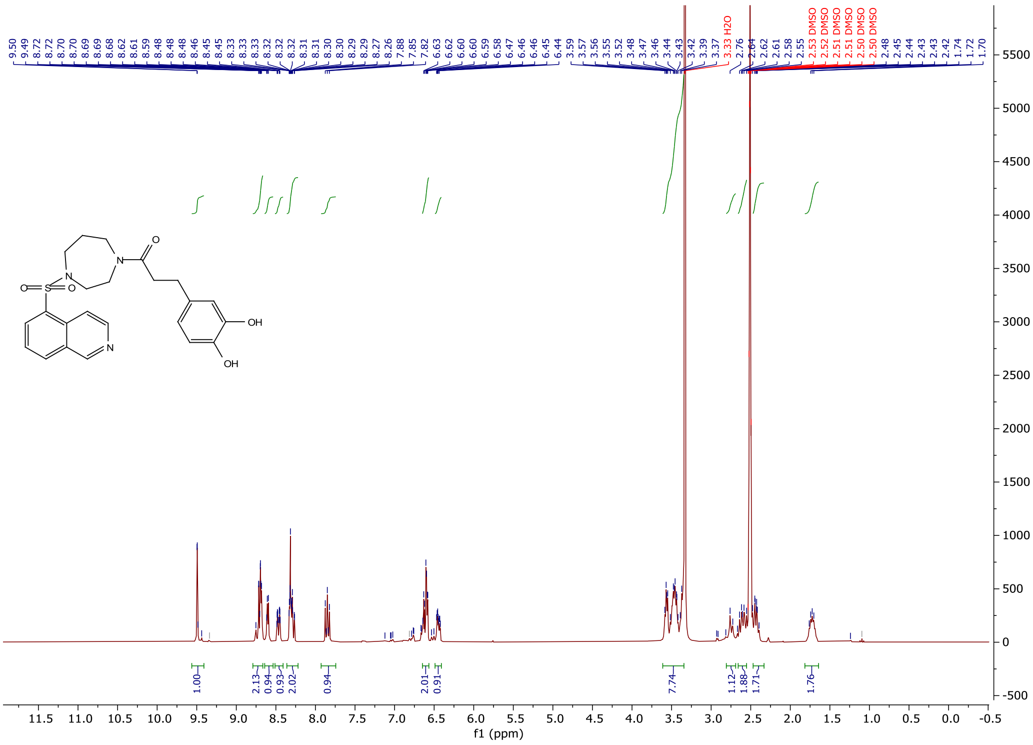

^{13}C-RMN

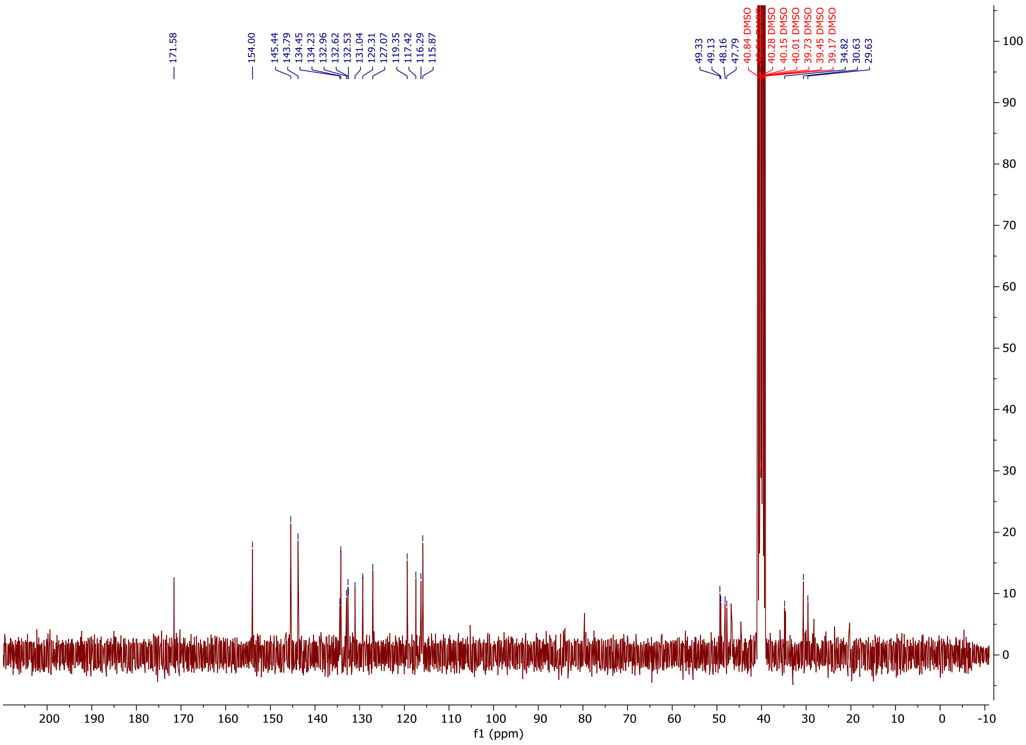

(*E*)-3-(4-hydroxy-3-methoxyphenyl)-1-(4-(isoquinolin-5-ylsulfonyl)piperazin-1-yl)prop-2-en-1-one (1e)

¹H-RMN

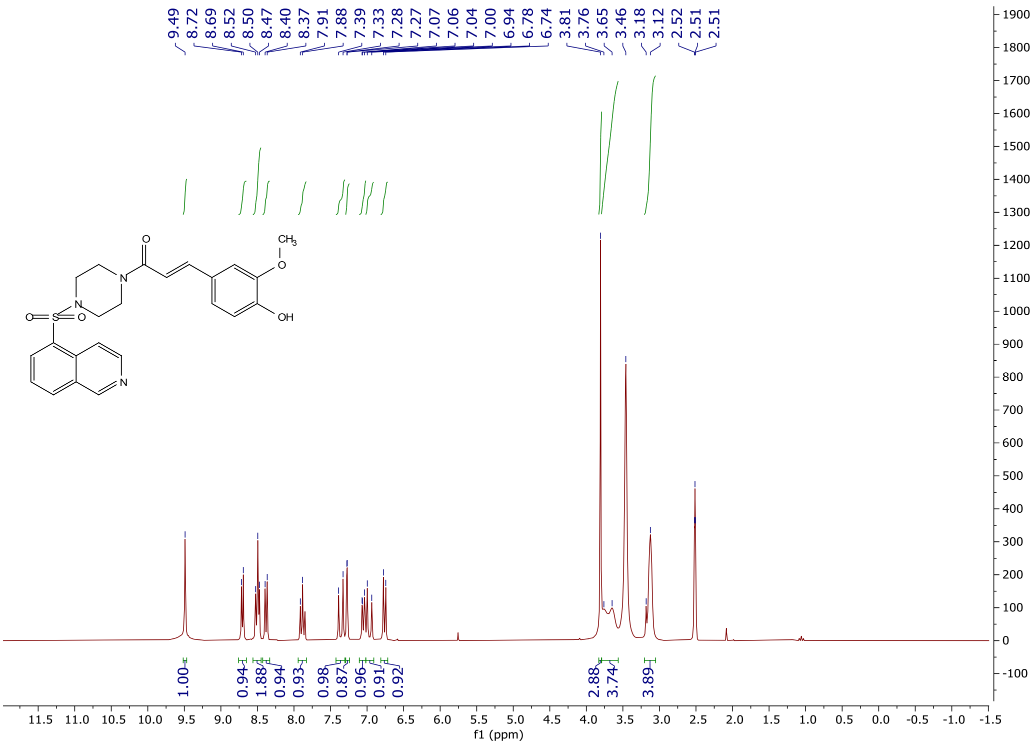

¹³C-RMN

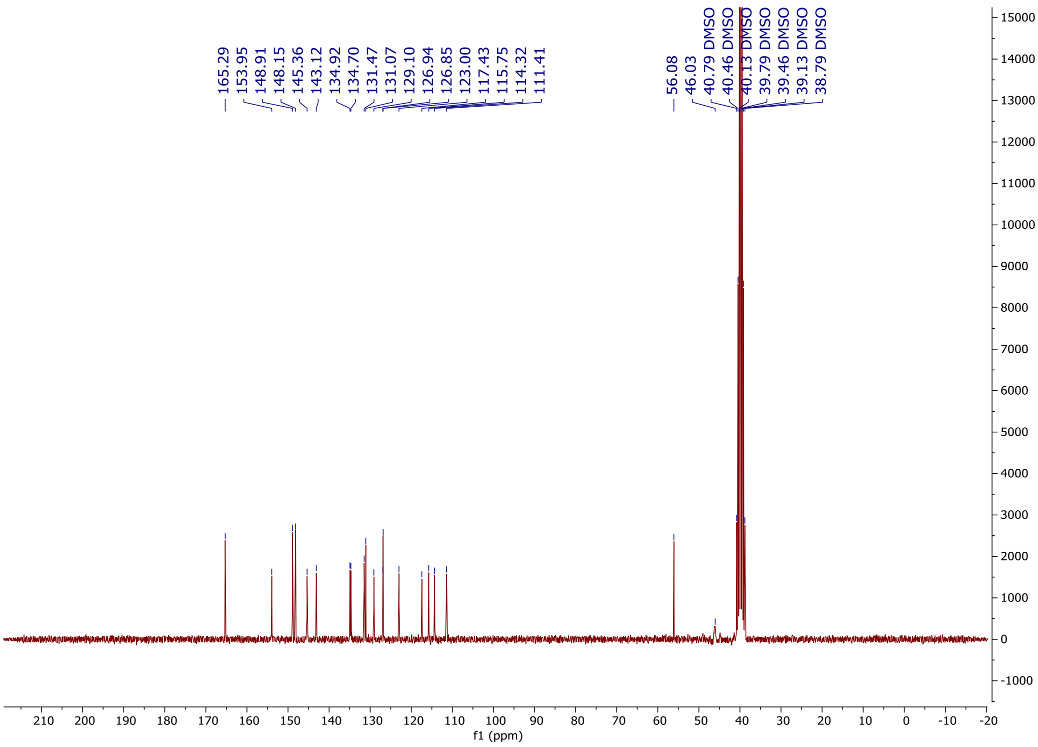

(*E*)-3-(4-hydroxy-3-methoxyphenyl)-1-(4-(isoquinolin-5-ylsulfonyl)-1,4-diazepan-1-yl)prop-2-en-1-one (1f)

^{1}H-RMN

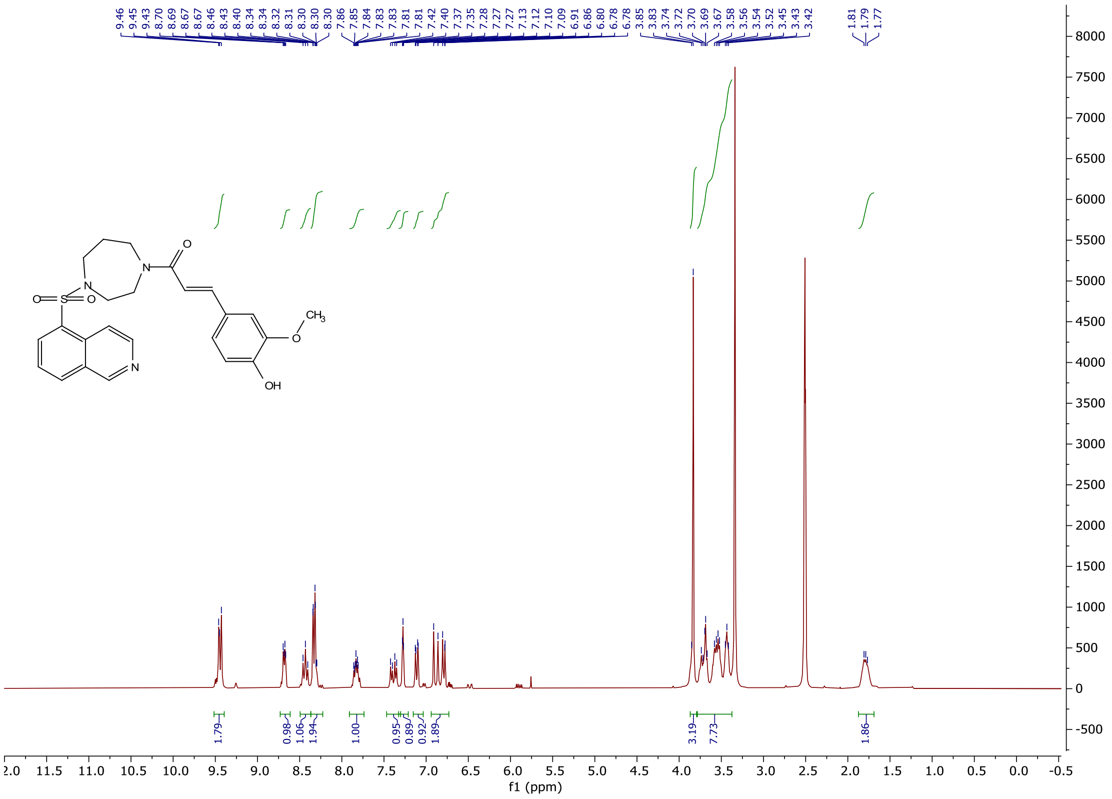

^{13}C-RMN

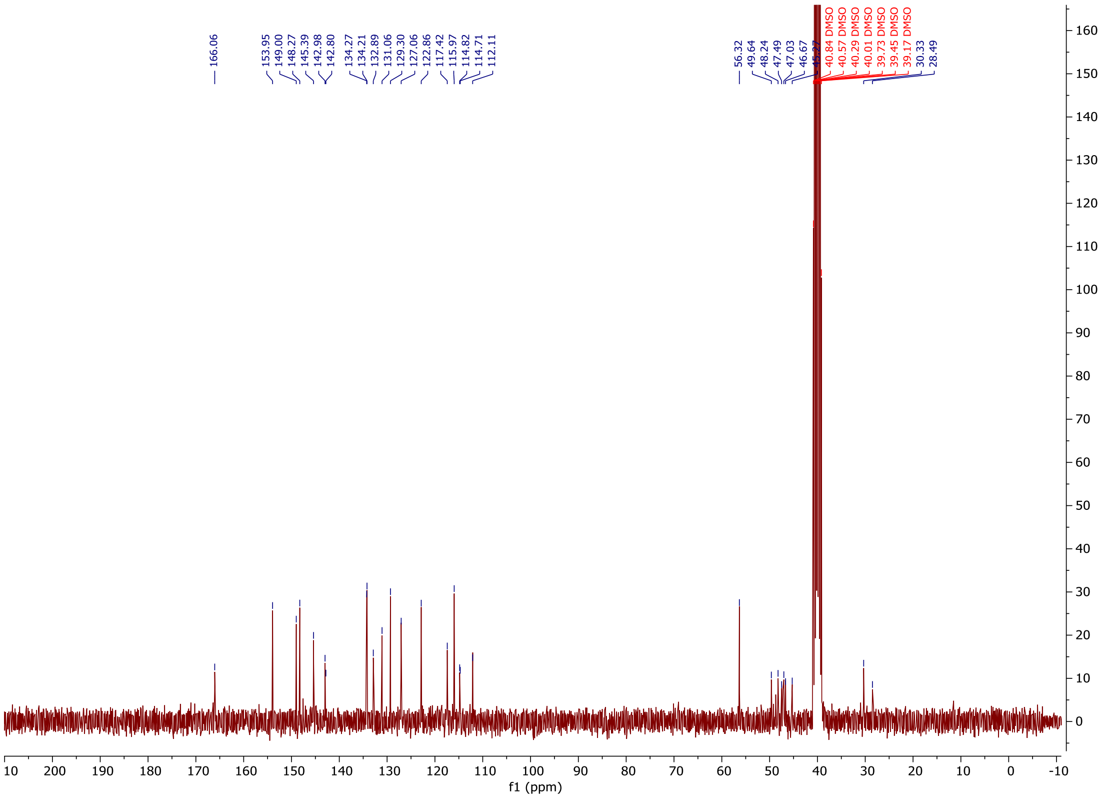

3-(4-Hydroxy-3-methoxyphenyl)-1-(4-(isoquinolin-5-ylsulfonyl)piperazin-1-yl)propan-1-one (1g)

^{1}H-RMN

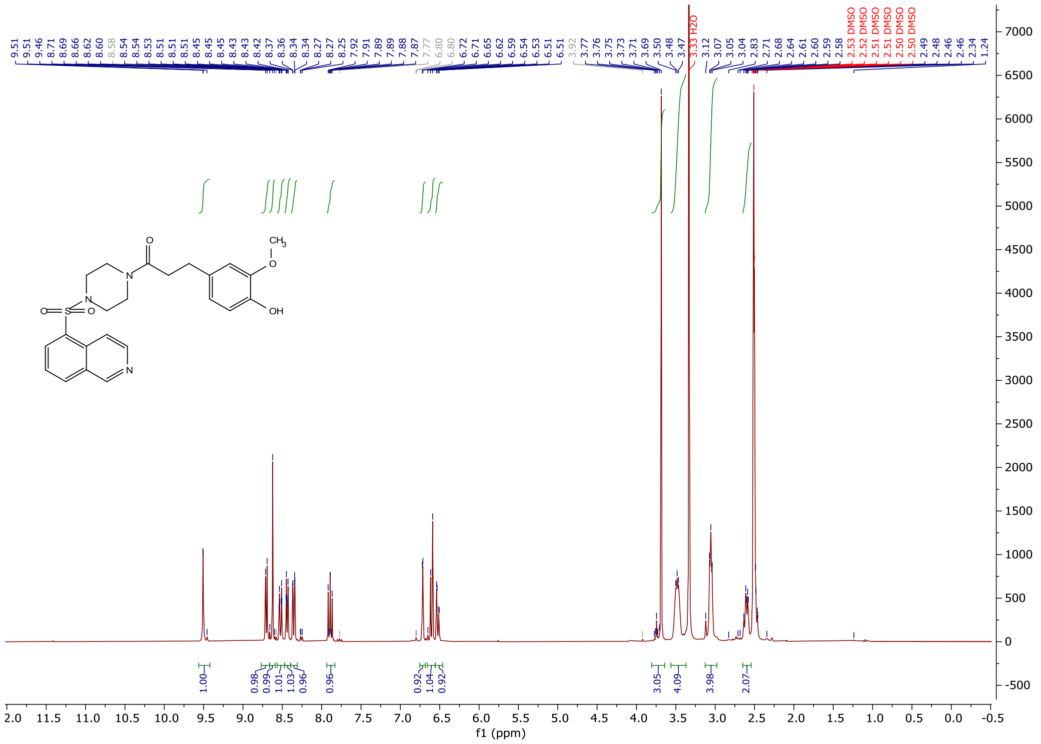

^{13}C-RMN

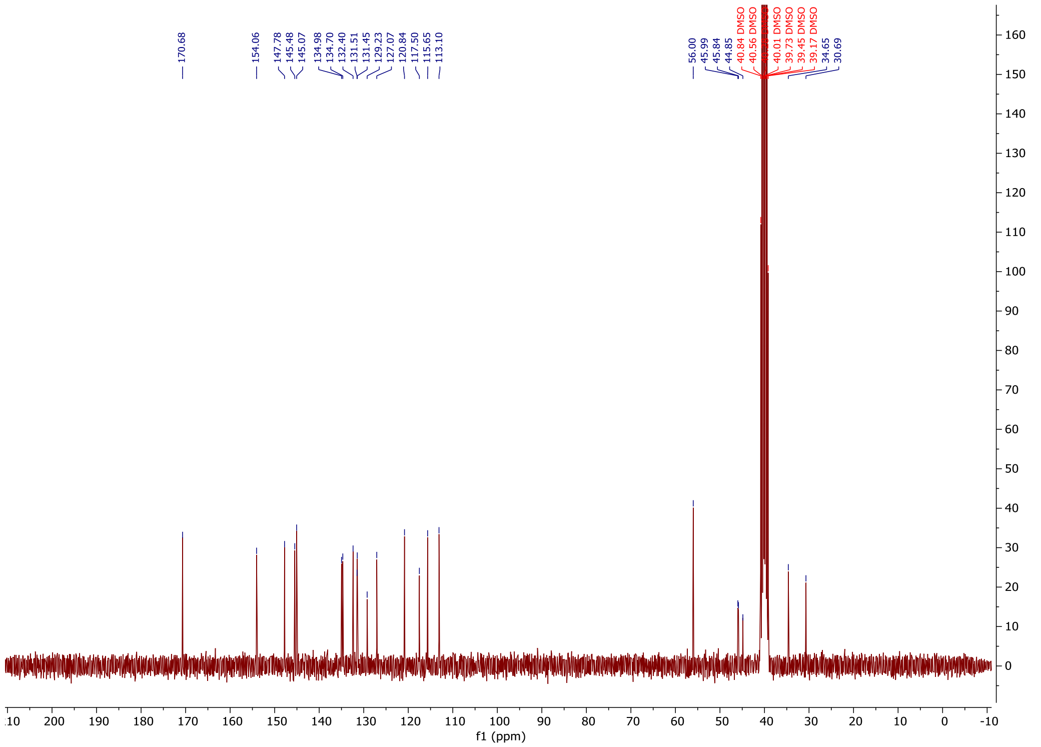

3-(4-Hydroxy-3-methoxyphenyl)-1-(4-(isoquinolin-5-ylsulfonyl)-1,4-diazepan-1-yl)propan-1-one (1h)

^{1}H-RMN

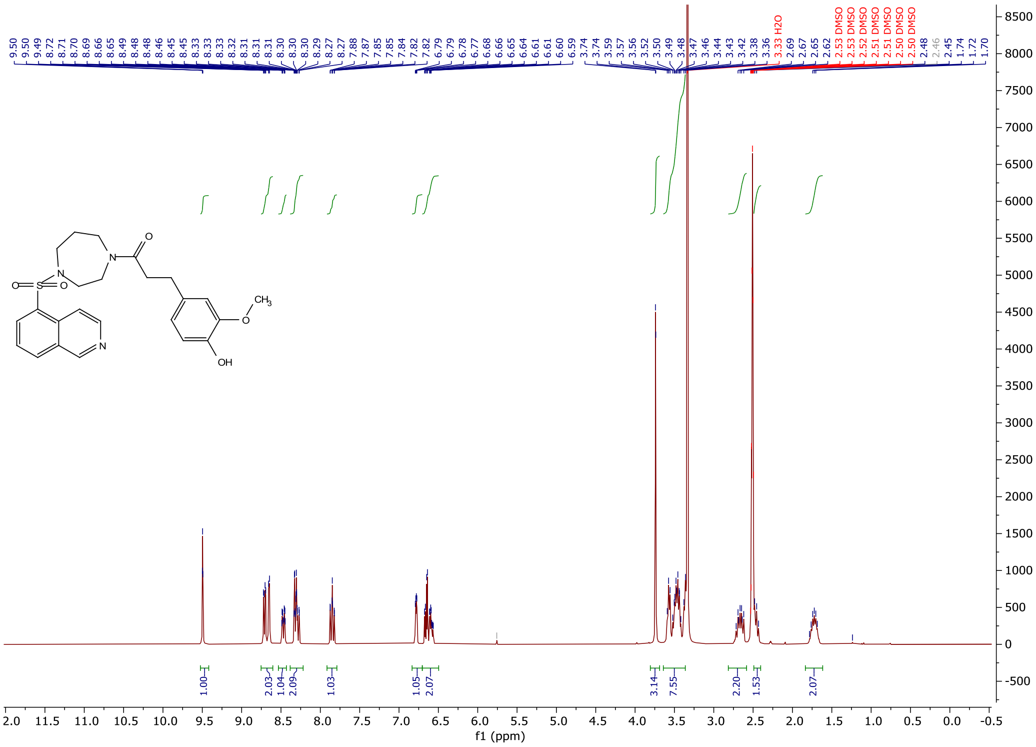

^{13}C-RMN

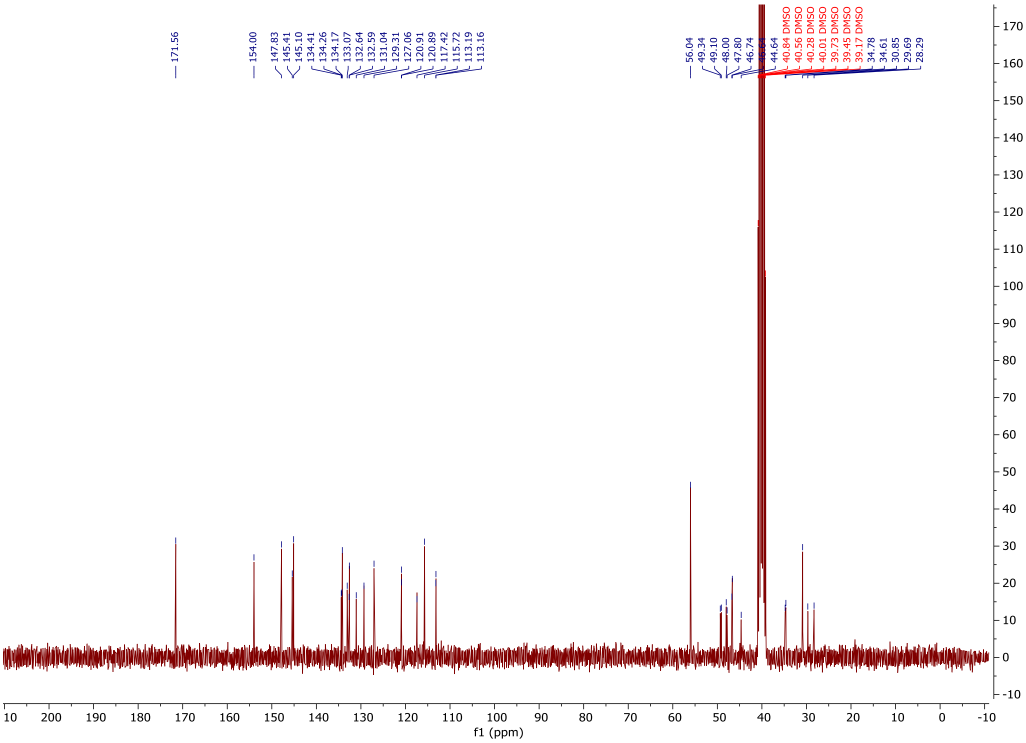

Copies of representative HPLC chromatograms

Compound 1c

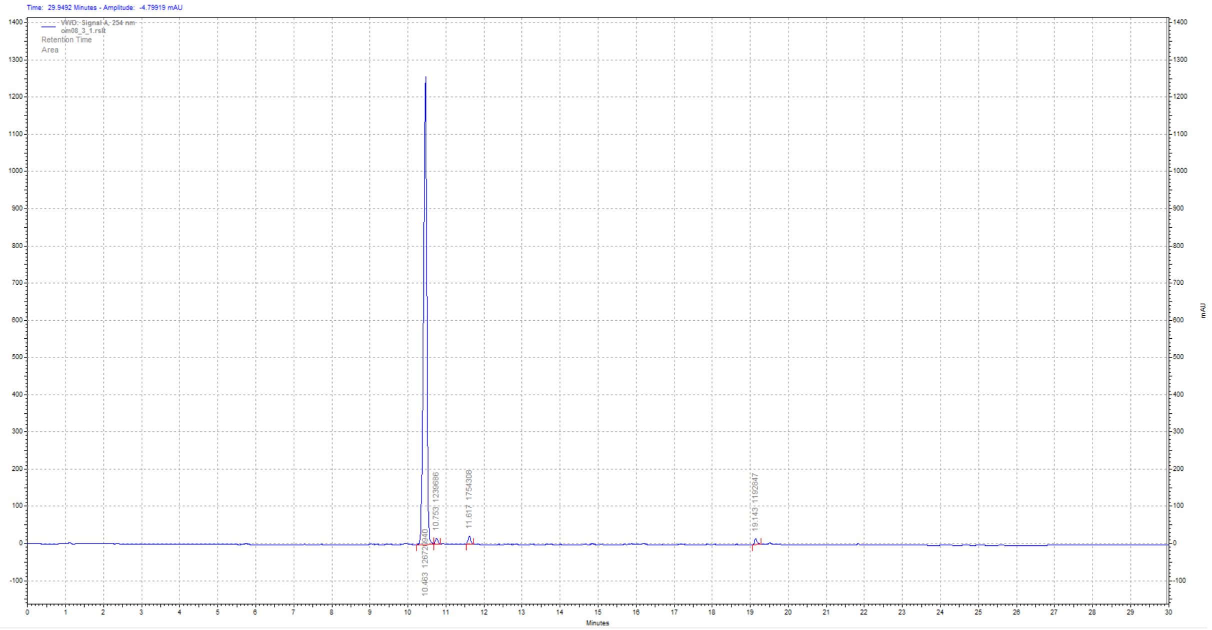

Compound 1d

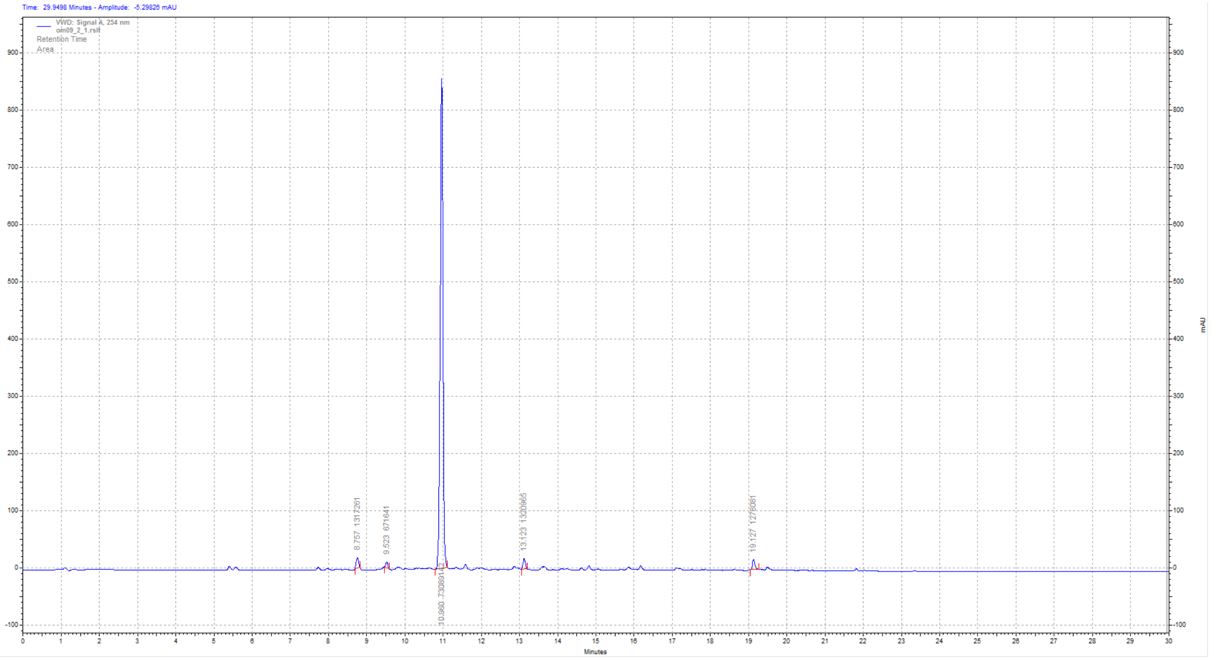

Compound 1e

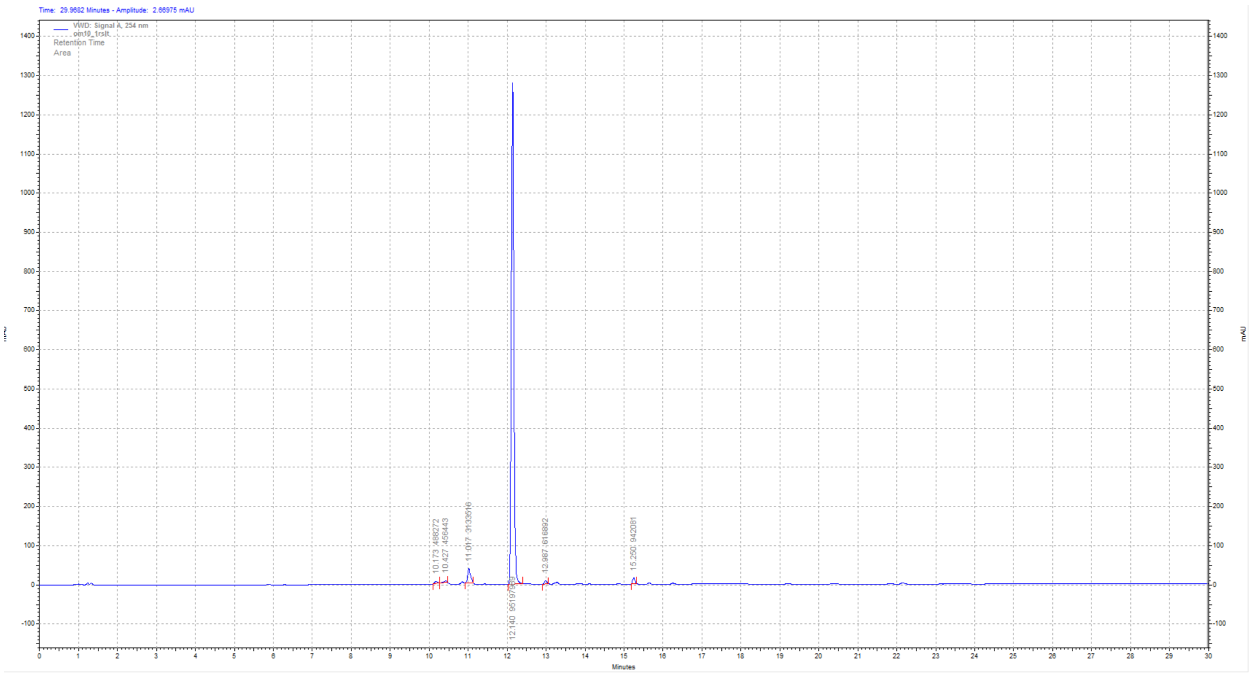

Compound 1f

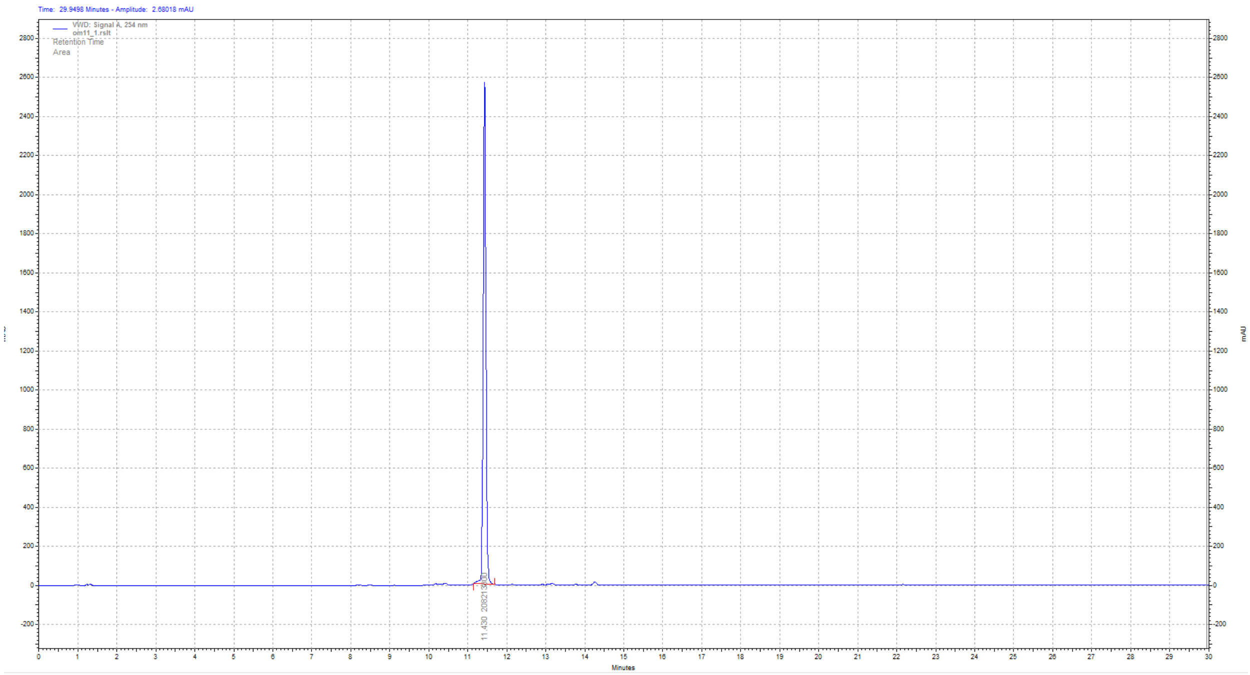

REFERENCES

1 Lipinski, C. A.; Lombardo, F.; Dominy, B. W.; Feeney, P. J. Experimental and computational approaches to estimate solubility and permeability in drug discovery and development settings. *Adv. Drug Deliv. Rev.* **2001**, *46*, 3-26.

2 Saina, A.; Zoete, V. A. A BOILED-egg to predict gastrointestinal absorption and brain penetration of small molecules. *Chem. Med. Chem.* **2016**, *11*, 1117-1121.

3 Baell, J.B.; Holloway G. A. New substructure filters for removal of pan assay interference compounds (PAINS) from screening libraries and for their exclusion in bioassays. *J. Med. Chem.* **2010**, *53*, 2719-2740.

4 Etrl, P.; Rohde, B.; Selzer, P. Fast calculation of molecular polar surface area as a sum of fragment-based contributions and its application to the prediction of drug transport properties. *J. Med. Chem.* **2000**, *43*, 3714-3717.

5 Delaney, J. S. Prediction of aqueous solubility and partition coefficient optimized by a genetic algorithm-based descriptor selection method. *J. Chem. Inf.* Model. **2004**, *44*, 1000-1005.
